# The Effects of Suspended Sediments, Dye, and Their Variation on Fish Behavior

**DOI:** 10.1002/ece3.74157

**Published:** 2026-08-13

**Authors:** Hannah M. Anderson, Emily Elsasser, James B. Barnett, Sigal Balshine

**Affiliations:** ^1^ Department of Psychology, Neuroscience & Behaviour McMaster University Hamilton Ontario Canada; ^2^ School of Natural Sciences Trinity College Dublin Dublin Ireland

**Keywords:** aquatic ecosystems, environmental fluctuation, fish behavior, turbidity, visual clarity

## Abstract

As human activities intensify, many freshwater environments are becoming darker. Suspended sediments released by human‐mediated erosion and manufactured dyes discharged into waterways act as pollutants that alter the visual environment. These substances absorb and refract light, reducing visibility levels and potentially interfering with animal behaviors such as predator avoidance, foraging, and social organization. Predicting the effects of such visual pollutants in the environment is complicated by the temporal variability of pollutant levels due to factors such as rainfall and wastewater flow. Here, we compared how an exposure to stable versus fluctuating levels of suspended sediments and dye respectively altered the social, movement, and anxiety‐like behaviors of captive zebrafish ( *Danio rerio* ). We found that fish moved more when exposed to fluctuating dye concentrations but decreased their movement when exposed to a stable suspended sediment concentration. Aggression decreased under stable exposures to both pollutants, but fluctuating conditions counteracted these effects. In contrast, shoaling behavior increased when fish were exposed to either pollutant regardless of fluctuation. This suggests that zebrafish may have different movement strategies for moving through turbid and dyed environments and that low visibility likely increases zebrafish shoaling. We also performed an optomotor response (OMR) assay to assess zebrafish visual perception under the different experimental conditions. While our OMR assays did not reveal any clear effects of either pollutant on visual perception, the fish performed fewer anxiety‐like behaviors during the low visibility OMR assays. This suggests that low visibility may reduce stress during stressful events. Overall, our results reveal that visual pollution influences the behavior of fish, and that both the variability and type of pollutant influence behavioral responses.

## Graphical abstract

Human activities are affecting the visual environments of freshwater ecosystems by introducing visual pollutants such as artificial dyes and elevated suspended sediment levels. Our research shows that dyes and suspended sediments can have contrasting effects of fish behavior, with dye increasing and sediments decreasing movement. We also found that fluctuating visual conditions can mediate the effects of low visibility on fish behavior.

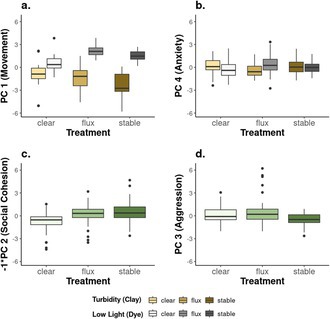

## 1 Introduction

Only 24% of Earth's terrestrial areas remain as undeveloped wilderness areas (Li et al. 2022), with nearly 10% of wilderness estimated to have been lost between 1990 and 2015 (Watson et al. 2016). Although most human activity is centered on land, freshwater ecosystems are thought to be even more vulnerable to the deleterious effects of human development than either marine or terrestrial ecosystems (World Wildlife Fund 2024). This vulnerability is due in large part to the clustering of human population centers near freshwater habitats, the tendency of human pollutants to collect in freshwater watersheds, and human use of freshwater habitats for activities such as drinking water, recreation, and industry (Williamson et al. 2008). Much of the research and conservation efforts centered on freshwater ecosystems focus on the physiological impacts of chemical pollutants such as bisphenol A (Muhamad et al. 2016), pesticides (Siemering et al. 2003), or fertilizer runoff (Pericherla et al. 2020). However, pollutants can also impact the visual environment, which can be just as serious as the effects of chemical pollution due to their ability to disrupt important life functions such as camouflage (Delhey and Peters 2017), foraging (Kjelland et al. 2015), and mate choice (Seehausen et al. 1997; Dominoni et al. 2020).

Two such visual pollutants affecting freshwater ecosystems are suspended sediments and man‐made dyes. Suspended sediments are a natural phenomenon that can be intensified by human‐mediated erosion (McLennan 1993; Syvitski and Kettner 2011; Vercruysse and Grabowski 2019). Man‐made dyes are artificial, water‐soluble coloring agents (Tkaczyk et al. 2020). Dyes primarily enter aquatic ecosystems through the wastewater of textile plants (Maheshwari et al. 2021), and to a lesser extent through food, cosmetics, and pharmaceutical manufacturing (Borgerding and Hites 1994). Suspended sediments and dyes impair visibility via different mechanisms: suspended sediments primarily scatter light as it enters the aquatic environment, creating a cloudy, masking effect sometimes known as turbidity (Fouilloux et al. 2022), while dyes primarily absorb light, reducing overall ambient light levels and altering the light spectrum (Fouilloux et al. 2022; Sandström 1999). The exact effects of these visual pollutants on the visual environment vary based on their makeup. For suspended sediments, the size, shape, and molecular composition of the sediments drive both their degree of light scattering and light absorption (Davies‐Colley and Smith 2001) while for dyes, the color of the component materials largely determines their range of light absorption (Tkaczyk et al. 2020). Whether these differences in visual conditions also result in differences in the behavior of animals living in these environments remains largely unknown (Fouilloux et al. 2022).

The effects of suspended sediments and turbidity on ecosystems are increasingly well studied, though the observed effects are highly variable across species and ecosystems (Henley et al. 2000; Kemp et al. 2011; Rodrigues et al. 2023). For example, walleye ( *Sander vitreus* ) showed increased biomass and foraging success under turbid conditions (Bristow et al. 1996; Tunney et al. 2018) but turbidity decreased foraging efficiency in three‐spined stickleback ( *Gasterosteus aculeatus* ; Sohel et al. 2017) and largemouth bass ( *Micropterus salmoides* ; Huenemann et al. 2012). Similarly, adult zebrafish ( *Danio rerio* ) exposed to periodic turbidity showed a decreased ability to discriminate visual stimuli (Suriyampola et al. 2018), but juvenile cuttlefish ( *Sepia officinalis* ) exposed to fluctuating turbidity showed increased capacity for visual discrimination (Goerger et al. 2021). These mixed results make broad generalizations about the effects of suspended sediments on behavior difficult.

In comparison to the research on suspended sediments, the visual impacts of dye on aquatic systems have received little study (Cuthbert et al. 2024). However, dyes are actively and increasingly used for environmental management (Cuthbert et al. 2024; Hussner et al. 2017) and are prevalent in the environment (Tkaczyk et al. 2020). The few existing studies suggest that light absorption by dye only minimally affects the growth rates of walleye, sunshine bass ( *Morone chrysops* × *M. saxatilis* ), and channel catfish ( *Ictalurus punctatus* ; Bristow et al. 1996; Ludwig et al. 2010; Tucker and Mischke 2020) and has little impact on the antipredator behavior of leopard frog tadpoles ( *Lithobates sphenocephalus* ; Bartson et al. 2018). Unfortunately, little else is known about how dye affects animal behavior (Cuthbert et al. 2024).

In the vast majority of suspended sediments and dyes impact research, animals have been exposed to stable concentrations (Gray et al. 2012; Sutherland and Meyer 2007; Chamberlain and Ioannou 2019), but in the real world environmental conditions are rarely stable (Gillain 2005; Kumar and Bahadur 2009; Loperfido et al. 2010). For example, rain tends to wash sediments into the water, especially in areas of active human development (Dethier et al. 2022; Vercruysse and Grabowski 2019; Wilkinson and McElroy 2007), which results in an initial increase in suspended sediment levels followed by a decline once the rain ends as sediments either fall out of suspension or are washed downstream (Gillain 2005; Loperfido et al. 2010; Navratil et al. 2012). Little is currently known about environmental concentrations of dye, either on average or over time (Tkaczyk et al. 2020). However, given that dyes primarily enter waters through wastewater (Maheshwari et al. 2021; Borgerding and Hites 1994), dye concentrations are likely to change based on wastewater flow. For example, dyes used in industrial production are likely to increase in concentration during work hours, when industrial wastewater flow is highest (Camacho‐Muñoz et al. 2014a, 2014b), while dyes found in personal care products like shampoo or cosmetics are likely to increase in the morning and evening before and after common work hours (Camacho‐Muñoz et al. 2014a; Li et al. 2018).

Fluctuating environmental conditions are generally considered to be more difficult to adapt to compared to stable conditions (Beauregard et al. 2013; Lawson et al. 2015; Morash et al. 2021). Thus animals that live in fluctuating environments require traits such as broad tolerance thresholds (da Silva et al. 2019) or the ability to quickly adjust trait expression as environmental conditions change (DeWitt et al. 1998). Results from studies using stable exposure regimes are therefore likely to underestimate natural effects. While some suspended sediment studies have allowed for changes in turbidity as the sediments settled out of suspension over time (Borner et al. 2015; Goerger et al. 2021; Van de Meutter et al. 2005), controlled studies examining the effects of exposure to fluctuating levels of suspended sediments or dye have not been attempted by other researchers.

Visual pollution from suspended sediments and dyes is particularly relevant for fishes because most known fish species rely on vision for tasks such as predator avoidance (Kelley and Magurran 2003; Kimbell and Morrell 2016), navigation (Braithwaite and De Perera 2006; Odling‐Smee and Braithwaite 2003), and foraging (Howe et al. 2018; Utne‐Palm 2002). Fishes also represent the most speciose group of vertebrates (Lévêque et al. 2008; Miller et al. 2018; Parravicini et al. 2013; Vasconcelos et al. 2007) and perform many important ecological roles such as maintaining local biodiversity (Mccauley et al. 2014), bioturbation of settled sediments (Adámek and Maršálek 2013; Oliveira Junior et al. 2019), and providing a key food source (Willson and Halupka 1995). Understanding the effects of suspended sediments and dye on fishes under both stable and fluctuating exposure regimes is therefore important for understanding the overall impacts of visual pollutants on aquatic ecosystems and their inhabitants.

We used zebrafish in our visual pollutant experiments because they are a social (Miller and Gerlai 2012; Suriyampola et al. 2015), visually‐oriented (Bilotta and Saszik 2001), and extremely well‐studied fish species (Bilotta and Saszik 2001; Bradford et al. 2011; Goldsmith 2004; Kalueff et al. 2013). Zebrafish originate from south Asia, a region subject to annual seasonal monsoons that result in recurrent fluctuations in suspended sediments and dramatic changes to visibility from the heavy rains (Arunachalam et al. 2013; Clift and Jonell 2021; Panda et al. 2011; Suriyampola et al. 2015). We hypothesized that evolving in such an environment would likely mean that zebrafish have a host of behavioral and physiological adaptations to fluctuating visibility. Zebrafish vision can also be readily measured in a laboratory setting using existing experimental methods (Bak‐Coleman et al. 2015; Krauss and Neumeyer 2003; LeFauve et al. 2021; Maaswinkel and Li 2003). In combination, these traits make zebrafish an informative reference and study organism for understanding how visual pollution affects a fish species well‐adapted to visual fluctuation.

In this study, we compared the effects of stable and fluctuating levels of (1) suspended sediments and (2) black dye on zebrafish behavior and visual perception. Specifically, we evaluated the social behavior, movement, space use, and ability to detect moving visual stimuli of zebrafish in clear water, in water with consistently low visibility, and in water with fluctuating visual conditions. We made the following predictions. (1) Low visibility would increase anxiety, resulting in the fish slowing down due to their reduced ability to see potential danger. We further predicted that the fish would spend more time near the walls or displaying agitated behavior indicative of stress under low visibility. (2) Low visibility would decrease social behaviors because fish would have an impaired ability to see each other and would therefore interact less with nearby conspecifics. (3) Suspended sediments and black pond dye would reduce zebrafish's ability to visually discriminate and respond to moving stimuli. (4) Dye would have a stronger impact on fish behavior compared to suspended sediments because zebrafish did not evolve in an environment containing artificial dyes. And finally, (5) fluctuating visibility would be harder to adapt to than stable visibility and therefore any observed effects of low visibility on fish behavior or visual discrimination would be stronger under fluctuating conditions compared to stable conditions.

## 2 Methods

### 2.1 Subjects and Housing

We used adult zebrafish purchased from the Zebrafish Breeding Colony at the University of Guelph on June 21, 2023. Before purchase, fish were raised under uniform laboratory conditions in glass aquaria with glass pebble substrate and fed with a combination of brine shrimp and daphnia. After purchase, fish were maintained in a 386.4 L polyethylene plastic oval stock tank (Tuff Stuff Products, Terra Bella CA, USA) at room temperature (21.5°C–23.0°C). Preceding and during Experiment 1, we maintained a 12.5:11.5 light:dark cycle with light levels slowly increasing from 9:00–9:30 AM and fading slowly off from 9:00–9:30 PM to simulate dawn and dusk. Due to a technical error in programming the lighting system, preceding and during Experiment 2 the room shifted to a 12:12 light:dark cycle with lights fading on from 8:00–8:30 AM and fading off from 7:30–8:00 PM, resulting in an extra 30 min of daylight during Experiment 1 compared to Experiment 2. Light was provided using a combination of Philips LED tubes (16713T8/MAS/48–840/1F2/P/DIM 10/1; Koninkliike Philips N.V., Amsterdam, Netherlands) and Philips Fluorescent168 tubes (F32T8/TL835; Koninkliike Philips N.V., Amsterdam, Netherlands), with a room illuminance of 796 lx (as measured from the center of the room). Fish were fed TetraMin Tropical Flakes (Spectrum Brands Pet LLC, Blacksburg VA, USA) until satiation once daily for 6 days a week prior to the experiments and every day during experiments.

### 2.2 Experimental Tanks

We used the same experimental tanks and testing arenas for both experiments. Fish were housed in 29.5 L (25 × 31 cm and 31 cm high) glass aquaria that also served as the experimental tanks. All tanks were supplied with air delivered via an airstone. Water was maintained at a depth of 8 cm to both minimize vertical movement during behavioral recordings and to ensure that fish could be easily observed even in low visibility treatments. In the wild, zebrafish are frequently found in shallow ponds and streams (Arunachalam et al. 2013; Spence et al. 2008), so this water depth was naturalistic. The sides of the experimental tanks were covered with opaque white plastic to increase the contrast while filming and to prevent visual interaction between neighboring tanks. The floors of all tanks were transparent glass devoid of substrate so we could film the fish from below. To maximize lighting, we mounted T5 LED shop lights (Barrina, Paris, France) directly above all tanks. Outside of filming, the tanks were covered with a thin transparent plastic lid.

### 2.3 Quantifying Visibility

#### 2.3.1 Turbidity Meter, Turbidity Tube, and Black Disc Test Measurements

We measured the approximate visibility of the suspended sediment and dye concentrations in our experimental treatments using a turbidity meter, a turbidity tube, and a modified black disc test. For the turbidity meter measurements, we used a HI98703 Turbidity Portable Meter (Hanna Instruments Inc., Smithfield RI, USA) in nephelometric turbidity units (NTU). Because suspended sediments primarily scatter light, we used the turbidity meter measurements when describing the suspended sediment treatments below. For the turbidity tube measurements, we used a 60 cm length turbidity tube (Dynamic Aqua Supply Ltd., Surrey BC, Canada), which is a clear, graded tube with a 4.3 cm diameter Secchi disc at the bottom. The turbidity tube quantified visibility by measuring the maximum vertical depth in centimeters at which the Secchi disc could be discerned by the human eye (Appendix S1, Table S1). Because dye primarily absorbs light rather than scatters it, a turbidity meter cannot accurately measure the visibility of dye conditions. We therefore use the turbidity tube measurements when describing the dye treatments below. For the black disc test, we used a modified version of the methods described by Kilroy and Biggs (2002). In short, we filled a 2.5 m long plexiglass water table with water containing dye or sediment that corresponded to the respective treatment conditions and measured the maximum horizontal distance (in centimeters) at which a 2 cm diameter matte black disc suspended on a rod was discernible by the human eye (Appendix S1, Table S2). The visibility measurement data is summarized in Appendix S1, Tables S1 and S2 (see Anderson et al. 2026a for full data).

#### 2.3.2 Spectrophotometry

To gain a more complete understanding of the visual environment within our treatment conditions we also measured the light absorbance of each treatment following the same methods outlined in our earlier research (Anderson, Qiu, et al. 2026). We used an Ocean Optics Flame S‐XR1‐ES (200–1025 nm) spectrophotometer with a DH‐Mini Deuterium Tungsten Halogen (200–2500 nm) light source passed through a plastic cuvette and recorded the spectrograms using OceanView (version 2.0.8.; Ocean Optics, Orlando FL, USA). We used OceanView's automatic calibration tool to calculate an integration time of 37,850 μs. We prepared three solutions equivalent to each of the treatment conditions (described below in Sections 2.4.1 and 2.4.2): high concentration clay (Experiment 1, stable and high phase flux conditions), moderate concentration clay (Experiment 1, low phase flux condition), high concentration dye (Experiment 2, stable and high phase flux conditions), and moderate concentration dye (Experiment 2, low phase flux condition). We used clear water as a reference sample. We sampled each treatment solution five times (*n* = 40 total samples) and averaged 20 scans per sample. While measuring these solutions with the spectrophotometer we simultaneously measured each solution with both the turbidity meter and turbidity tube for comparison (see Appendix S1, Table S1).

**FIGURE 1:**
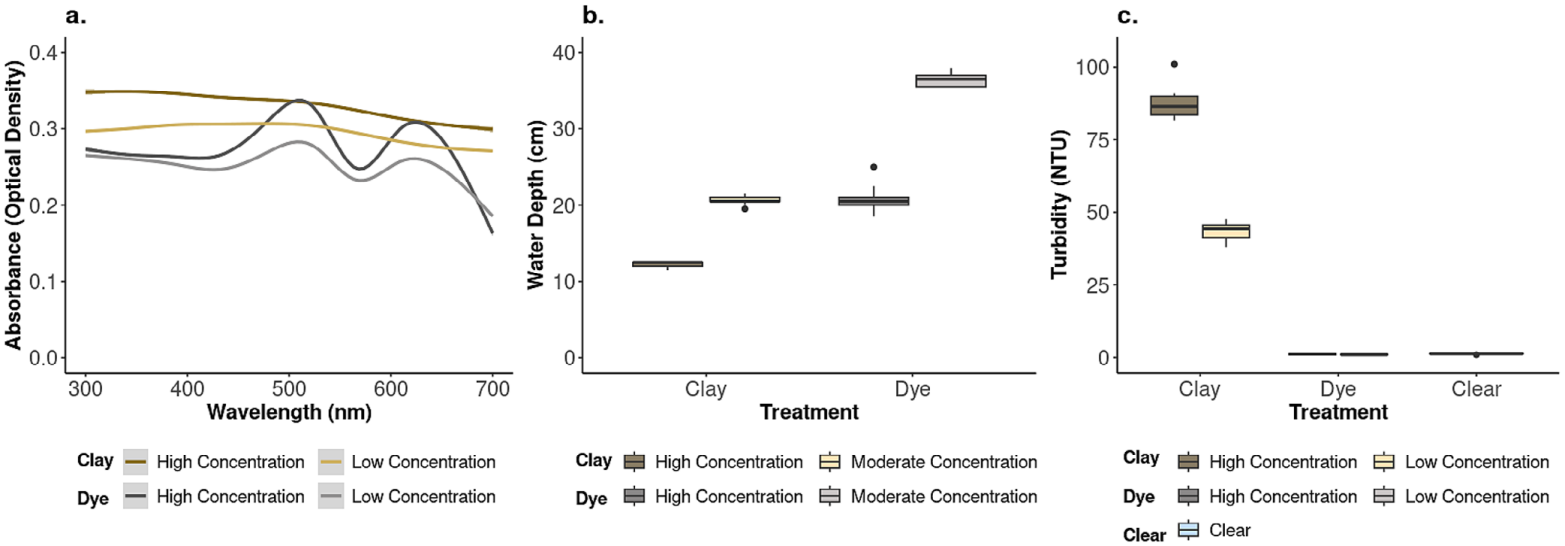
Visibility of all treatment conditions as measured by a spectrophotometer, a turbidity tube, and a turbidity meter. Across all plots the high clay concentration is represented by brown, the low clay concentration is represented by tan, the high dye concentration is represented by dark gray, and the low dye treatment concentration is represented by light gray. In (c), clear water is represented by light blue. The middle line of each box plot represents the median, the lower and upper hinges of each box respectively represent the first and third inter‐quartiles, and the upper and lower whiskers respectively extend to the largest and smallest datapoint within 1.5× the interquartile range of the corresponding hinge. (a) The absorbance spectra of each clay and dye treatment concentrations by optical density. The envelopes around each line represent the 95% confidence intervals. Both the clay treatments are largely flat, with the high concentration centered around 0.33 OD and the low concentration around 0.30 OD. Both dye treatments show absorbance peaks around 500 and 625 nm, which peak around 0.35 OD in the high concentration and 0.28 OD in the low concentration. (b) The maximum water depth (in cm) that a Secchi disc is visible under each clay and dye treatment concentrations as measured by the turbidity tube. The low dye concentration showed the greatest visibility with a median water depth of 36.5 cm, the high dye and low clay concentrations showed similar visibility, with median water depths of 20.5 cm, and the high clay concentration had the lowest visibility, with a median water depth of 12.5 cm. (c) The turbidity of each clay and dye treatment concentration and the clear water treatment as measured by the turbidity meter. The high clay concentration shows the highest turbidity levels with a median of 86.4 NTU. The low clay concentration shows the next highest turbidity with a median of 44.3 NTU. Both dye concentrations and the clear water treatment show similar turbidity values close to zero.

We processed the light absorbance spectra in R (version 4.4.1; R Core Team 2020) using the R package ‘pavo’ (version 2.9.0; Maia et al. 2019). To account for sensor noise, we averaged together all spectra from the same solution and smoothed the spectral curves using a span of 0.25. We plotted the spectra using the R package ‘ggplot2’ (version 3.5.1; Wickham 2016). The absorbance spectra of the clay treatment solutions were largely flat, with an average optical density (OD) of 0.33 ± 0.0006 for the high clay concentration (turbidity ± standard error, 88.0 NTU ± 1.8) and 0.30 ± 0.0035 for the moderate clay concentration (43.3 ± 1.0 NTU; Figure 1). The absorbance spectra for both dye treatments showed two similar peaks (Figure 1), one around 500 nm (green/cyan; high dye concentration 0.35 OD, moderate dye concentration 0.28 OD) and one around 625 nm (orange/red; high dye concentration 0.34 OD, moderate dye concentration 0.28 OD). Overall, the average OD for the high dye concentration (maximum visibile depth, 20.9 ± 0.6 cm) was 0.28 ± 0.0010 and the average OD for the moderate dye concentration (36.4 ± 0.3 cm) was 0.25 ± 0.0005.

### 2.4 Experimental Protocols

#### 2.4.1 Experiment 1: Clay Turbidity Exposure

Fish were maintained in a large laboratory stock tank as described above for 64 days before the start of the experiment. On August 24, 2023 we created 30 mixed sex shoals consisting of two males and two females (four fish per shoal with a 1:1 male:female sex ratio), each chosen haphazardly from the stock tank (*n* = 120 total fish), placing only one shoal of four fish each per experimental tank. Zebrafish shoals in the wild vary widely in size from four to 300 fish, with small shoals more common in slow flowing and shallow habitats (Suriyampola et al. 2015), so our four fish groups were an environmentally relevant group size. Each shoal was randomly assigned to both an experimental tank and to a treatment. There were three treatments (described below) with 10 shoals per treatment. We weighed and photographed each fish during initial group creation. A single observer (EE) later used the digital photographs to measure the standard length of all fish in ImageJ (version 1.54f; Schneider et al. 2012). Average body size (mean body, 0.73 ± 0.01 g; mean standard length, 36.34 ± 0.31 mm) did not vary across the three treatments (body mass, *F* = 0.26, degrees of freedom (df) = 2, *p* = 0.77; standard length, *F* = 0.41, df = 2, *p* = 0.67). We allowed fish to habituate to their experimental tanks for 1 day before starting each treatment exposure. During this habituation day we conducted a 50% water change at 10:00 AM and performed the same stirring regime (described below) used during experimental treatments. Including the habituation day, the experiment occurred over 5 days: 1 day of habituation, 2 days of standardized behavioral recordings (described in Section 2.5), and 2 days of visual perception assays (described in Section 2.6), with the clay exposure occurring over a total of 4 days.

Our treatment conditions were a clear water control, a stable high suspended sediment level, and a condition that fluctuated between the high suspended sediment level in the morning and a low suspended sediment level in the afternoon. For brevity, we will refer to these as the clear, stable, and flux conditions throughout the remainder of the manuscript. To create suspended sediments we used kaolin clay (Amson Naturals, Richmond Hill ON, Canada). Kaolin clay is considered safe for use with fish and is frequently used in suspended sediments and turbidity studies (Chamberlain and Ioannou 2019; Suriyampola et al. 2018). In the clear condition no clay was added during the experiment (*n* = 8 samples, 0.92 ± 0.11 NTU). In the stable condition we maintained a constant, high level of clay throughout the experiment using the methods described below (*n* = 8 samples, 65.24 ± 2.04 NTU). In the flux condition the turbidity fluctuated between a high turbidity level equivalent to the stable treatment (*n* = 4 samples, 64.19 ± 1.35 NTU) in the morning and a lower turbidity level (*n* = 4 samples, 37.05 ± 3.30 NTU) in the afternoon. We will refer to the high clay concentration of the flux treatment as high phase flux and the low clay concentration of the flux treatment as low phase flux throughout the remainder of the manuscript. For some ecological context, water under 4 NTU in a drinking glass would appear perfectly clear to the naked human eye (World Health Organization 2017) and a typically clear stream can easily reach turbidity levels of at least 65 NTU following rainfall (Loperfido et al. 2010). Little information exists on the turbidity levels in the natural range of zebrafish (Arunachalam et al. 2013; Engeszer et al. 2007), although there is evidence that zebrafish habitat can reach at least 20 NTU after the monsoon season ends when rainfall is relatively low (Morgan et al. 2019). Hence turbidity levels of 65 NTU like the ones used here are highly plausible.

To maintain sediment levels, we performed two 50% water changes per day across all treatments: one in the morning (10:00 AM) and one in the afternoon (4:00 PM). To create the suspended sediment treatments, we prepared the water and clay mixtures in separate containers until they reached the requisite turbidity level 1 h before each water change. During each water change, we first removed water from the experimental tanks, then re‐stirred the clay mixtures, and then added the mixtures to the corresponding tanks. For the stable condition, the clay mixture was added at each water change to ensure consistent turbidity. For the clear condition the water was prepared following the same methods to create the clay mixture except without adding any clay to the water. For the flux condition, the clay mixture was added in the morning, but clear water was added in the afternoon. Thirty minutes after each water change, we sampled the turbidity of two randomly chosen tanks per treatment (*n* = 6) to track potential changes in sediment levels. Because sediments settle out of water over time we stirred all tank chambers in a stereotyped zig‐zag pattern with a plastic spatula once an hour while the lights were on to maintain constant turbidity levels throughout the day. Our previous research has shown that stirring has minimal effect on overall zebrafish behavior (Anderson, Qiu, et al. 2026).

#### 2.4.2 Experiment 2: Dye Visibility Exposure

On September 21, 2023 we created another 30 mixed sex groups following the same methods as in Experiment 1 (10 shoals per treatment, two males and two females per shoal, 120 total fish). Fish had been returned to the laboratory stock tank between experiments, so it is possible that some of the fish from the first experiment were also used in the second experiment, although shoal composition during the two experiments would have been different. There were 28 days between the first and second experiments. The mean body mass of the fish in this experiment was 0.74 ± 0.01 g, with a mean standard length of 37.96 ± 0.25 mm. The average body size did not vary across treatments (body mass, *F* = 1.23, df = 2, *p* = 0.30; standard length, *F* = 0.11, df = 2, *p* = 0.90). The fish were again allowed to habituate for 1 day in their experimental chambers with the stirring procedure in place and one 50% water change conducted at 10:00 AM. As in the first experiment, this experiment occurred over 5 days, with 1 day of habituation, 2 days of standardized behavioral recordings, and 2 days of visual perceptions assays. The dye exposure occurred over the last 4 days of the experiment.

In this experiment we used black pond dye (Outdoor Water Solutions, Springdale AR, USA) to manipulate the visual environment instead of kaolin clay. Pond dye is commonly used to improve pond appearance and is considered nontoxic to fish (Outdoor Water Solutions 2008). Similar pond dyes have been used in other fish studies with no ill effects (Bristow et al. 1996; Tucker and Mischke 2020; Ward and Vaage 2019). For comparison with the Experiment 1 suspended sediment treatments, a kaolin clay value of 45 NTU on the turbidity meter is equivalent to a visibility measurement of ~20 cm on the turbidity tube, and a value of 90 NTU on the turbidity meter is equivalent to ~12 cm on the turbidity tube (Appendix S1, Table S1).

Unlike the turbidity meter, which only required a small sample of water (~10 mL), the turbidity tube required a much larger water sample (~284 mL) to measure visibility. As a consequence, we were unable to sample the visibility of the experimental chambers during the experiment as we did in Experiment 1 because this would impact the water volume in the tanks; however, during a previous study visibility did not change noticeably over 24 h when using dye (Anderson, Qiu, et al. 2026). We therefore used fixed concentrations of dye prepared following the same procedures for preparing the clay solutions in Experiment 1. All visibility measures listed below were piloted ahead of the experiment and were measured by a single observer under experimental lighting conditions (HMA; see Appendix S1, Table S1).

For the control condition no dye was added throughout the experiment (visibility > 60 cm). In the stable condition we maintained a constant, high level of dye (30 drops dye for every 28 L of water, ~54 μL dye/1 L water, or 20.89 ± 0.64 cm visibility, *n* = 9 samples). As in Experiment 1, the visibility in the flux condition fluctuated between a high dye amount equivalent to the stable condition in the morning (*n* = 9 samples, 20.89 ± 0.64 cm visibility) and a moderate dye amount in the afternoon (15 drops dye/28 L of water, ~27 μL dye/1 L water, *n* = 9 samples, 36.44 ± 0.31 cm visibility). In this experiment, the water changes occurred at 9:00 AM and 3:00 PM, with dye mixtures prepared prior to water changes instead of clay. Aside from these differences, treatment conditions were identical between this experiment and Experiment 1, including the stirring procedures. Information on dye concentrations in the environment are unfortunately limited (Tkaczyk et al. 2020), but visible distances below 40 cm in waters naturally colored brown by dissolved organic material have been observed in natural environments (Ranåker et al. 2012).

### 2.5 Standardized Behavioral Recordings

To sample the behavior of our fish under turbid (suspended sediment) and low light (pond dye) conditions, we video recorded the shoals on each of the first 2 days of the experiment an hour after each water change (11:00 AM and 5:00 PM in Experiment 1; 10:00 AM and 4:00 PM in Experiment 2; hence each shoal was recorded a total of four times). This schedule allowed for an equal number of recordings during the high and low phase flux conditions with two recordings per phase. Eight videos from the dye experiment (four each from the stable and flux conditions) were excluded from final analysis due to poor tracking by the tracking software TRex (see below; in the end 228 out of 236 total recorded videos were used in the analyses). We recorded fish behavior using Canon VIXIA HF R800 series cameras (Canon Inc., Tokyo, Japan) mounted vertically beneath the tanks. Each recording was 15 min long. We discarded the first 5 min of each recording to account for disturbance by the researchers while placing the camera, resulting in a total scored video length of 10 min. To increase visibility and contrast on the video recordings, we temporarily removed the airstones and replaced the transparent lids with opaque white lids during video recording.

We used the automated tracking program TRex (version 1.1.9; Walter and Couzin 2021) to track fish movement throughout our recordings. We manually calibrated the threshold value for detecting the fish against the background for each video using the inbuilt interface TGrabs and used the default mode averaging method to detect the background versus the fish. From TRex, we exported the *X*, *Y* coordinate values of the centroid (as defined by TRex) and orientation of all fish across all videos. We manually calibrated the distance values for all *X*, *Y* coordinates using a known distance. From the exported data for each video, we calculated measures for the whole shoal, including the mean speed, maximum speed, proportion of time spent active (activity), proportion of total tank space used (space use), mean within‐shoal variation in fish body angle (polarity), mean shoal area, mean neighbor distance, and mean proximity to the nearest wall (wall proximity).

**FIGURE 2:**
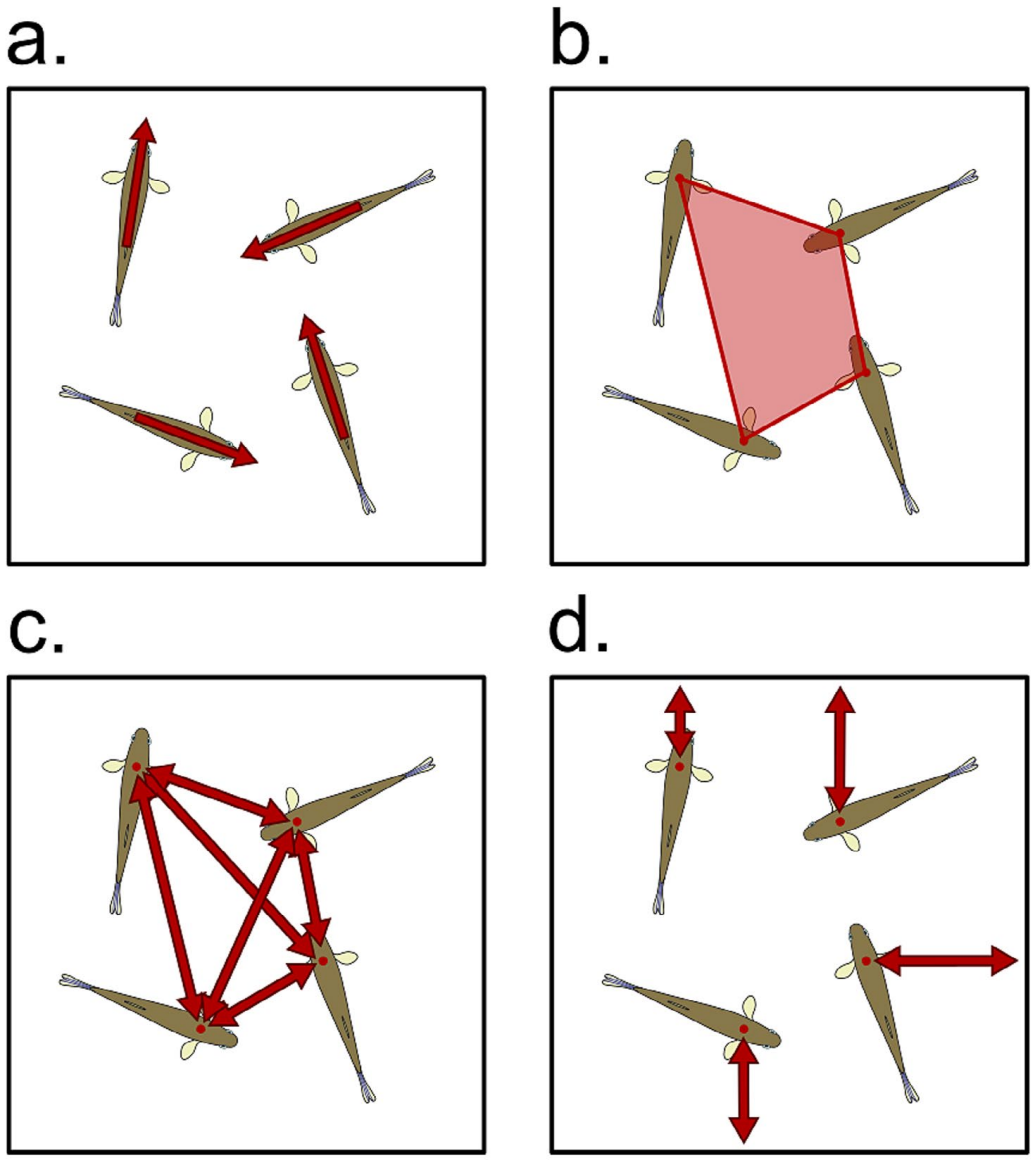
Illustration of the measurements for (a) polarity, (b) shoal area, (c) mean neighbor distance, and (d) wall proximity. (a) Polarity, where the forward‐facing angle of each fish is calculated in and then used to calculate the circular variance of the angles of all fish on a scale from zero (all fish facing opposite directions) to one (all fish facing the same direction). (b) Shoal area, where lines are drawn between the centroids of each fish (as calculated by TRex) and a polygon is created from the non‐crossing lines. The area of the polygon is used as the measurement of shoal area. (c) Mean neighbor distance, where lines are drawn between the centroids of each fish and the mean length of all lines calculated. (d) Wall proximity, where a line is drawn between the centroid of each fish and the closest point on the closest wall. The mean length of all lines is then calculated.

In brief, we calculated speed using the distance in centimeters that each fish moved per second (~30 frames/s) and then took the mean and maximum values for each shoal per recording. We calculated activity by first manually reviewing the videos to calibrate a threshold speed under which fish were not actively moving (< 1.5 cm/s). We then classified each second of fish movement as “active” if their speed was greater than our threshold value and “inactive” if their speed was under our threshold value. From this, we calculated the proportion of time the fish spent active in relation to the total scored video time (10 min). We calculated space use by dividing the experimental tank into a 20 × 24 grid where each grid cell was 1 cm^2^ and then calculated the proportion of grid cells that were occupied by the centroid of any fish for at least 1 s. We calculated polarity for each video by taking the average circular variance of the orientation of all fish in radians (Figure 2a). We calculated shoal area and mean neighbor distance by drawing lines between the centroids of each fish. For shoal area, we used the non‐crossing lines to create a polygon and then calculated the polygon's area for each frame. We then averaged the shoal area across the entire video (Figure 2b). For mean neighbor distance, we averaged the lengths of the lines between all fish across each frame and then averaged the distances across the entire video (Figure 2c). We calculated proximity to the nearest wall by drawing the shortest possible line between the centroid of each fish and the nearest wall and then averaging the lengths across all fish for the entire video (Figure 2d). TRex position tracking tends to inflate movement values, so for all of these measures we manually corrected for noise in TRex's outputs using rolling averages. The full code and explanation of calculations is published in Anderson et al. (2026b).

We also used Behavioral Observation Research Interactive Software (BORIS, version 8.21.5; Friard and Gamba 2016) to score three aggressive (chases, aggressive contacts, lateral displays), and one affiliative (follows) behavior (scored independently by three observers). The behaviors we scored were based on definitions described by Kalueff et al. (2013). In brief, we defined “chases” as the rapid pursuit of one fish by another fish where both fish exhibited rapid tail beating and stayed within 1–1.5 body lengths of each other. We defined “aggressive contacts” as any physical contact (except spawning) between two fish where one fish pulled away or flinched from the other. We defined “lateral displays” as two fish lining up parallel to each other in opposite directions so the head of one fish was opposite the tail of the other fish with both fish quivering. We defined “follows” similarly to chases except the movement was slower, with no rapid tail beating, and a possible distance between the lead and following fish of up to two body lengths.

### 2.6 Optomotor Response Assay

**FIGURE 3:**
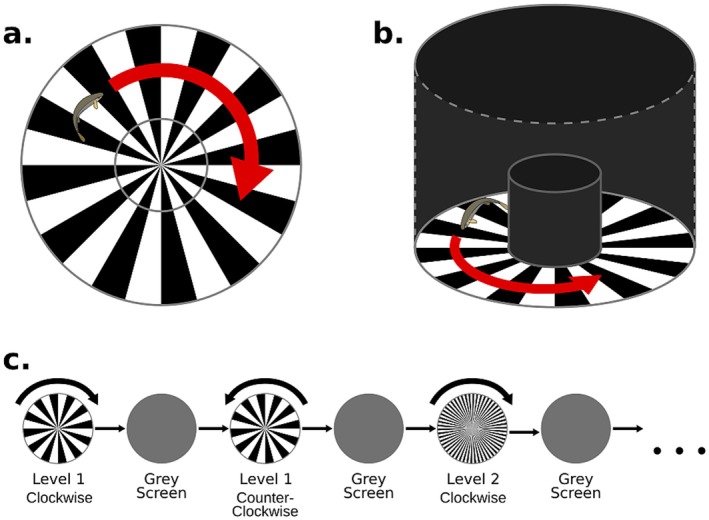
Layout and order of optomotor response assay. (a) A top‐down view of the optomotor response assay arena as it was filmed. (b) A cutout side view of the optomotor response arena. (c) The pattern of stimulus presentation, starting with the level 1 stimulus (24 alternating black and white wedges) rotating in the clockwise direction, followed by a neutral gray screen, then the level 1 stimulus rotating in the counterclockwise direction, and so on. There were six stimuli levels, with the wedges getting thinner and smaller at each subsequent level.

The optomotor response (OMR) is a behavior where animals orient themselves in response to, or physically pursue, a repetitive moving pattern (Maaswinkel and Li 2003; Naumann et al. 2016; Rock and Smith 1986). OMRs can be used to detect the spatiotemporal visual acuity of an individual by altering the spatial frequency of the repeating pattern and observing at which frequency the pattern becomes perceptually indistinguishable and the individual stops responding (Maaswinkel and Li 2003; Suriyampola et al. 2018). For both experiments, we used a variant of the zebrafish OMR assay developed by LeFauve et al. (2021) to measure the zebrafish's ability to visually discriminate moving stimuli under treatment conditions. We placed the fish in a circular, 17 cm diameter arena with opaque black walls. A 5.5 cm black PVC cylinder placed in the center of the arena created a circular track with a width of 5.5 cm. The floor of the arena was clear and placed on top of a Samsung T350 Series 22 Inch FHD 1080p Computer Monitor (Samsung Digital Group, Suwon, South Korea) from where we projected the assay stimuli (Figure 3). This monitor had a 75 Hz display, which is above the flicker‐fusion threshold for adult zebrafish (~50 Hz; Branchek 1984). The stimuli were produced through the PsychoPy (version 2023.1.2; Peirce et al. 2019) Python shell (version 3.8.10; Python Software Foundation 2021) using a customized version of the LeFauve et al. (2021) stimulus code (Anderson et al. 2026b).

We performed the OMR assays on the final 2 days of the suspended sediment and dye experiments (after the standardized behavioral recordings). Outside of the assays, fish remained in their original treatment conditions. We performed each OMR assay between the first and second water change of the day when the flux condition visibility was lowest (high phase flux) and comparable to the stable condition visibility level (high concentration sediment/dye levels). On the first OMR day (day three of exposure) we performed the OMR assays in clear water while on the second day (day four of treatments) we performed the OMR assays under low visibility conditions (Experiment 1, 91.1 ± 0.98 NTU; Experiment 2, 28.3 ± 0.25 cm). For Experiment 1 fish the low visibility in the OMR arena was created with kaolin clay and for the Experiment 2 fish the low visibility in the OMR arena was created with dye. Conditions between the 2 days were otherwise identical. On each day we randomly selected five shoals per treatment and haphazardly selected one male and one female from each of these shoals to be tested with the OMR assay. We ran each male/female fish pair through the OMR simultaneously using two parallel arenas, with sex per arena alternating every assay. This gave us a final sample size of 60 total fish per experiment: 30 fish per water clarity level (clear versus low visibility OMR assay), or 5 fish of each sex per treatment condition (clear, flux, or stable) per water clarity level (clear versus low visibility OMR assay).

All stimuli were comprised of a rotating circle made of alternating, equally sized black and white wedges (Figure 3). We presented six levels of stimuli over the course of each assay, with each subsequent level increasing in spatial frequency so that the wedges became thinner and consequently more difficult to visually perceive. The level 1 stimulus wedges were the easiest to perceive and level 6 stimuli were the most difficult to perceive. At the first level the stimulus was comprised of 12 pairs of contrasting black and white wedges (24 total wedges). For each subsequent stimulus difficulty level, the number of black and white wedge pairs increased by 42 (or 84 total wedges), which resulted in thinner and smaller wedges as the stimulus level increased. In order, each stimulus level was comprised of 24, 108, 192, 276, 360, and 444 total wedges respectively. We controlled the speed of the stimulus rotation so that the speed of the leading edge of the wedges stayed constant as wedge number increased.

To avoid any potential biases in stimulus directionality we presented each stimulus level twice, the first time rotating clockwise for 20 s and the second time rotating counterclockwise for 20 s, before moving to the next stimulus level. To limit fatigue and break up stimulus presentations, we displayed a gray screen for 20 s between each stimulus presentation. A single OMR assay would therefore consist of a clockwise stimulus at level 1 for 20 s, then a gray screen for 20 s, then a counterclockwise stimulus at level 1 for 20 s, then a gray screen for 20 s, then a clockwise stimulus at level 2, and so on, until counterclockwise stimulus level 6 was reached (Figure 3). All assays were filmed from above using the same cameras as used for the standardized behavioral recordings described in Section 2.5 above (Canon Inc., Tokyo, Japan). Before the start of each OMR assay, the focal fish was placed in the arena and given 5 min to habituate.

All OMR assays were manually scored by a single observer (EE) in BORIS (Friard and Gamba 2016). We scored two OMR behaviors (positive OMR and negative OMR). Following the definitions provided by Maaswinkel and Li (2003), we defined “positive OMR” as a fish swimming in the same direction as the rotation of the stimulus for at least half the arena track. We defined “negative OMR” as a fish swimming in the opposite direction as the rotation of the stimulus for at least half the arena track. We recorded the duration of positive OMRs and negative OMRs at each stimulus level. Though OMR research typically focuses only on positive OMRs (Krauss and Neumeyer 2003; Maaswinkel and Li 2003), some evidence suggests adult zebrafish are more likely to perform negative OMRs when the visual stimulus is displayed on the floor of the tank (Bak‐Coleman et al. 2015; Gore et al. 2024), so we scored both. We also scored two anxiety‐like behaviors (jumps and agitation behavior). We defined “jumps” as a fish propelling itself out of the water. We defined “agitation behavior” as disordered, jerky movement performed in seemingly random directions. We recorded the number of jumps and agitation behaviors and whether they occurred during a stimulus presentation or during an interstitial gray screen.

### 2.7 Statistical Analyses

We conducted all statistical analyses in R (version 4.4.1; R Core Team 2020) using a significance (alpha) value of 0.05. We created all plots using the R package “ggplot2” (version 3.5.1; Wickham 2016). We fit all statistical models using the function ‘glmmTMB’ from the R package ‘glmmTMB’ (version 1.1.9; Brooks et al. 2017) and visually inspected the fit of all models by plotting the output of the function ‘simulateResiduals’ from the R package ‘DHARMa’ (version 0.4.6; Hartig 2022). We used the ‘emmeans’ and ‘emtrends’ functions from the R package ‘emmeans’ to calculate estimated marginal means, confidence intervals, and conduct any relevant post hoc analyses (version 1.10.3; Lenth 2024). We also used the emmeans package's default Tukey method to adjust for multiple comparisons during post hoc analyses. For significant main effects of predictors with more than two levels we conducted post hoc pairwise comparisons across all factor levels and for significant interactions we performed post hoc pairwise comparisons of the predictor of primary interest (treatment) at each level of the interacting predictor variable(s). In the case of overlapping significance (e.g., if the main effect of treatment and the interaction between treatment and time were both significant) we only conducted post hoc analyses on the highest order interaction. See Anderson et al. (2026b) for code and detailed explanations of all statistical analyses.

#### 2.7.1 Standardized Behavioral Recordings

We measured 12 different behaviors in our standardized behavioral recordings. In order to reduce the number of multiple comparisons required, we reduced the dimensions of our data into representative principal components (PCs) with a principal component analysis (PCA) using the base R function “prcomp” (R Core Team 2020). We centered and scaled all data within prcomp before analysis. We decided on the number of PCs to include in our final analyses by examining the weights on each PC and using the PCs that best captured our research questions while maximizing the captured variance (Appendix S1, Table S3).

In total, we used four PCs which collectively captured 70% of the total variance (see Appendix S1, Table S4). PC 1 (36% of total variance) was primarily weighted by activity, space use, and mean swimming speed, with all three measures increasing as PC 1 increased. Therefore, we considered PC 1 to primarily be a measure of fish movement, with greater PC 1 values indicating greater movement. We used PC 1 to test part of our first prediction, that fish would move less in low visibility conditions. PC 2 (13% of total variance) was primarily weighted by shoal area and mean neighbor distance, with both measures increasing as PC 2 increased. Therefore, we consider PC 2 to primarily be a measure of social cohesion. Since smaller values of shoal area and mean neighbor distance indicate greater social cohesion (a tighter shoal), smaller values of PC 2 also indicate greater social cohesion. PC 3 (12% of total variance) was primarily weighted by aggressive contacts, chases, and lateral displays, with all three measures increasing as PC 3 increased. Therefore, we consider PC 3 to primarily be a measure of aggression, with greater PC 3 values indicating greater aggression. We used PCs 2 and 3 to test different aspects of our second prediction, that fish would reduce their social interactions in low visibility conditions. PC 4 (9% of total variance) was primarily weighted with maximum speed and proximity to the nearest wall, with maximum speed increasing and distance to the nearest wall decreasing as PC 4 increased. Since rapid movement in zebrafish is generally the result of darting and/or erratic movement, which are considered to be anxiety‐caused behaviors (Kalueff et al. 2013), and proximity to the nearest wall in an open arena is a common measure of anxiety in zebrafish (Godwin et al. 2012), we consider PC 4 to primarily be a measure of anxiety. Since greater values of maximum speed (faster darting) and smaller values of distance to the nearest wall are generally indicative of greater anxiety, larger values of PC 4 indicate greater levels of anxiety‐like behavior. We used PC 4 to test part of our first prediction, that fish would display more anxious behavior in low visibility conditions.

We fit each PC with a linear mixed model. We included treatment (clear, stable, and flux), experiment (clay and dye), time of day (morning and afternoon), and all of their interactions as fixed effects and shoal ID as a random effect to account for repeated measures.

The use of PCs in our primary statistical analyses necessarily combines and simplifies the data in ways that may make the individual behavioral components more difficult to interpret. To improve clarity on how individual behaviors changed across treatments we also fit linear models to four representative behaviors that fell within each of our PCs. The behaviors selected had high loadings, were conceptually representative of their respective principal component, and thus were practical to interpret. PC 1 was a measurement of fish movement, so we selected mean speed as a representative behavior. For PC 2 (social cohesion) we chose mean neighbor distance; for PC 3 (aggression) we chose aggressive contacts; and for PC 4 (anxiety) we chose proximity to the nearest wall. In brief, we fit mean speed, mean neighbor distance, and wall proximity with linear mixed models with the same factors as for the PC analyses listed above. We fit aggressive contacts with a generalized linear mixed model using a Poisson distribution and the same factors as the previously listed models. The full methods and results of these models have been included in Appendix S4 and largely support the results of our PC analyses reported below, though see Appendix S4 and Figure S8 for a full discussion of these analyses.

#### 2.7.2 Optomotor Response Assay

To analyze the responses of the fish to the OMR assay we separately fit the total duration of positive and negative OMRs across two generalized linear mixed models using a Tweedie distribution as implemented in ‘glmmTMB’, with the power parameter restricted to 1 < power < 2 and following the methods outlined in Dunn and Smyth (2008). We included the experimental tank treatment condition (clear, clay flux, dye flux, clay stable, and dye stable), water clarity during the OMR assay (clear and low visibility), the logarithm of stimulus level (1–6, corresponding to the increasing spatial frequency of the wedges on the spinning disc), sex (male versus female), and all possible two‐way interactions as fixed effects, with shoal ID as a random effect to account for repeated measures. To analyze anxiety during the OMR assay we also fit the total number of anxiety‐like behaviors (sum of the number of jumps and agitation behaviors) to a generalized linear mixed model with a Poisson distribution. We included treatment (clear, clay flux, dye flux, clay stable, and dye stable), water clarity during the assay (clear and low visibility), the display on the screen (stimulus and gray screen), sex (male and female) and all possible two‐way interactions as fixed effects and shoal ID as a random effect to account for repeated measures. We only evaluated two‐way interactions to minimize the number of contrasts.

Our model fit tests detected a potentially poor fit of the logarithm of stimulus level as a fixed effect in our negative OMR model, so we also conducted a sensitivity test to verify overall good model fit. Our sensitivity test verified that our results were robust to alternative model fits (see Appendix S1, Table S5), so we present the results of the original model here.

### 2.8 Ethics

All experimental procedures were assessed and approved by the Animal Research Ethics Board of McMaster University (Animal Utilization Protocol No. 22‐03‐09) and adhere to the guidelines set by the Canadian Council on Animal Care (CCAC) Guidelines for the use of animals in research. All fish were carefully monitored throughout and following the experiments for any signs of injury or distress. No signs of distress or injury were observed due to experimental conditions, and fish returned to normal behavior within 1 to 2 days upon return to the stock tank.

## 3 Results

### 3.1 Behavioral Recordings

#### 3.1.1 Movement (Principal Component 1)

Across all the treatments, the fish were active for ~80% of the behavioral recording time (mean activity ± standard error; 0.78 ± 0.02; range 0–1), had a mean speed of 5.67 ± 0.15 cm/s, and used ~75% of their tank space (space use ± standard error; 0.75 ± 0.01; range 0–1).

**FIGURE 4:**
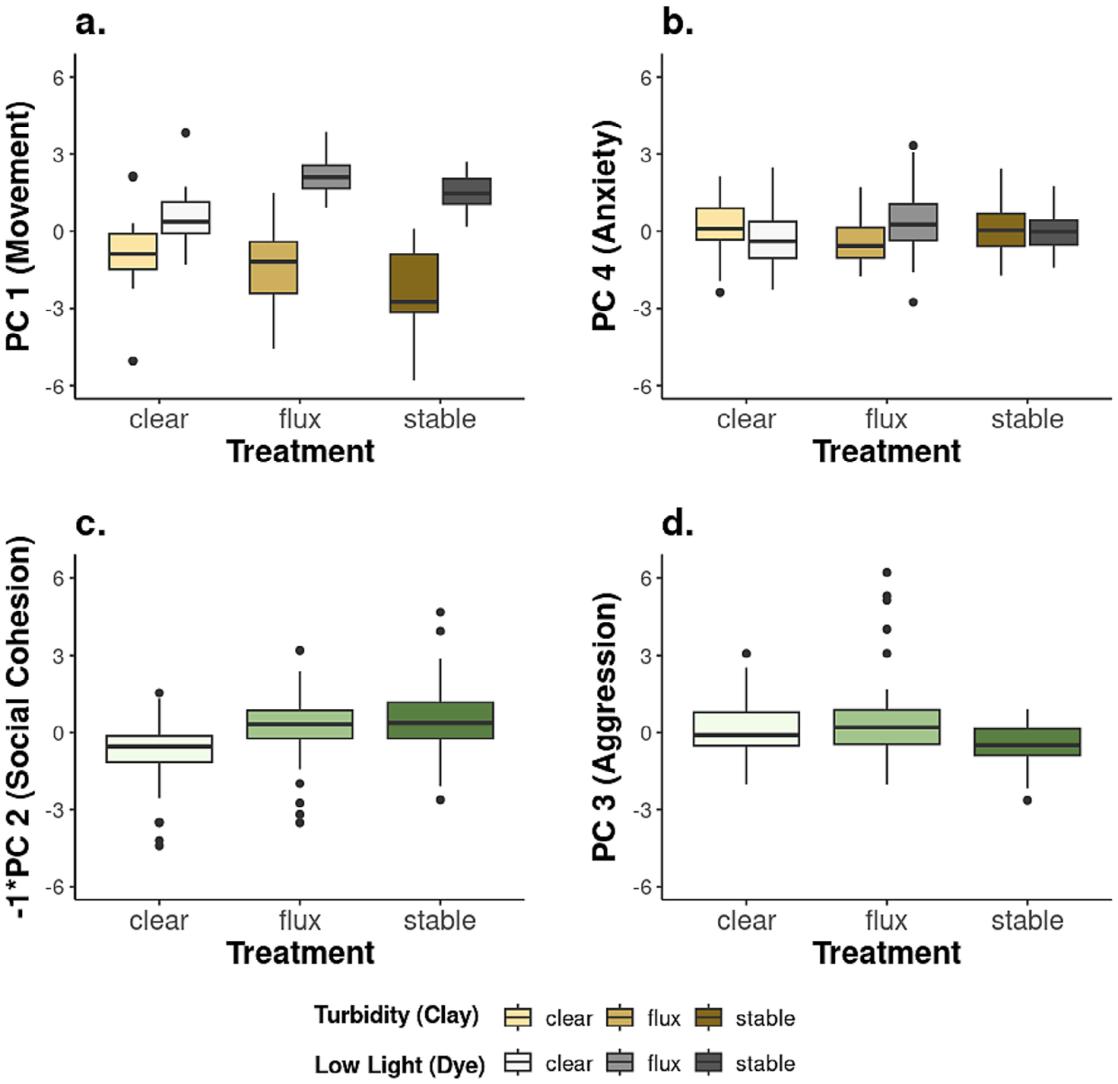
Significant effects on (a) fish movement, (b) anxiety, (c) social cohesion, and (d) aggression (PC 3). The middle line of each box plot represents the median, the lower and upper hinges respectively represent the first and third inter‐quartiles, and the upper and lower whiskers respectively extend to the largest and smallest datapoint within 1.5× the interquartile range of the corresponding hinge. For figures (a) and (b), Experiment 1 (clay) is represented by shades of tan and Experiment 2 (dye) is represented by shades of gray. The treatments are represented by the shades of each color, with the lightest shade representing the clear treatment, the middle shade representing the flux treatment, and the darkest shade representing the stable treatment. For figures (c) and (d) the treatments are represented by different shades of green, with the lightest shade representing the clear treatment, the middle shade representing the flux treatment, and the darkest shade representing the stable treatment. (a) Fish movement patterns (as captured by PC 1) in the morning across both experiments and all three treatments. Increasing values of PC 1 indicate increasing levels of fish movement (percent of time spent active, percent of tank space used, and mean swimming speeds). (b) Fish anxiety patterns (as captured by PC 4) across both experiments and all three treatments. Increasing values of PC 4 indicate increasing levels of anxiety‐like behaviors (proximity to nearest wall and maximum speed). (c) Fish social cohesion patterns (as captured by PC 2) across all three treatments. Social cohesion is presented here as −1 × PC 2 so that increasing values on the y‐axis represent increasing values of social cohesion (shoal area and mean neighbor distance). (d) Fish aggression patterns (as captured by PC 3) across all three treatments indicate increasing levels of aggression (aggressive contacts, lateral displays, and chases).

We detected a three‐way interaction between all three factors in our model (overall model: treatment × experiment × time of day, *χ* ^2^ = 8.29, df = 2, *p* = 0.016, Appendix S2, Figure S1). The fish in the stable suspended sediments treatment moved *less* compared to fish in the clear treatment, though only in the morning (post hoc pairwise comparison: clay stable × clear, *t*‐ratio = 2.50, df = 213, *p* = 0.035; Figure 4a and Appendix S2, Table S7). On average, the fish in the stable suspended sediments treatment were 6% slower, 3% less active, and used 15% less tank space compared to the fish in clear treatment in the morning. In contrast to the suspended sediments experiment, the fish moved more in the flux dye treatment compared to the clear treatment, though again only in the morning (post hoc pairwise comparison: flux dye × clear, *t*‐ratio = −3.38, df = 213, *p* = 0.003, Figure 4a and Appendix S2, Table S7). On average, the fish in the flux dye treatment were 59% faster, 17% more active, and used 4% more tank space compared to the fish in the clear treatment in the morning. All other post hoc comparisons within this three‐way interaction were nonsignificant (see Appendix S2, Table S7).

The fact that we only observed these effects of clay and dye in the morning, when the flux and stable treatments had the same poor visibility level, suggests that these are true effects of fluctuating visibility levels and not merely a side effect of differences in average visibility between the flux and stable treatments.

#### 3.1.2 Social Cohesion (Principal Component 2)

Across all three treatments and both experiments the mean shoal area was 34.50 ± 0.78 cm^2^ and the mean neighbor distance was 10.29 ± 0.10 cm. Fish were equally socially cohesive in the suspended sediments and dye experiments (Table 1). We detected a significant effect of treatment (*χ* ^2^ = 20.66, df = 2, *p* < 0.001, Table 1 and Appendix S2, Figure S2 and Table S6) where fish shoals were overall tighter (more socially cohesive) in the stable and flux treatments compared to the clear treatment (post hoc comparison: stable × clear, *t*‐ratio = 4.19, df = 213, *p* < 0.001; post hoc comparison: flux × clear, *t*‐ratio = 3.63, df = 213, *p* = 0.001; Figure 4c and Appendix S2, Table S7), though there was no significant difference between the flux and stable treatments (Appendix S2, Table S7). Shoal area was on average 18% smaller and mean neighbor distance 7% shorter in the stable treatment compared to the clear treatment, and shoal area was 11% smaller and mean neighbor distance 5% shorter in the flux treatment compared to the clear treatment. This means that, overall, fish social cohesion was greater when visibility was poor (stable and flux treatments) compared to when visibility was high (clear treatment).

**TABLE 1:** Statistical results for the linear mixed models of tested principal components. In the first column, “treatment” refers to the visual treatment condition (clear, stable, flux), “experiment” refers to the visual pollutant (suspended sediments, dye), and “time” refers to the time of day of the recording (morning, afternoon). The Wald *Χ* ^2^ value in the second column is the test statistic. “df” in the third column stands for “degrees of freedom.” The fourth column is the *p*‐value, where bold font indicates *p* < 0.05.

Term	Wald *Χ* ^2^	df	*p*
PC 1, Movement			
Treatment	5.57	2	0.06
Experiment	**118.96**	**1**	**< 0.001**
Time	**3.86**	**1**	**0.0496**
Treatment × experiment	**6.81**	**2**	**0.03**
Treatment × time	2.81	2	0.25
Experiment × time	0.08	1	0.78
treatment × experiment × time	**8.29**	**2**	**0.02**
PC 2, Affiliation			
Treatment	**20.66**	**2**	**< 0.001**
Experiment	0.01	1	0.93
Time	**6.23**	**1**	**0.01**
Treatment × experiment	1.47	2	0.48
Treatment × time	3.98	2	0.14
Experiment × time	0.00	1	0.96
Treatment × experiment × time	3.19	2	0.20
PC 3, Aggression			
Treatment	**13.55**	**2**	**0.001**
Experiment	2.58	1	0.11
Time	2.43	1	0.12
Treatment × experiment	1.65	2	0.44
Treatment × time	0.27	2	0.87
Experiment × time	0.11	1	0.74
Treatment × experiment × time	0.68	2	0.71
PC 4, Anxiety			
Treatment	0.40	2	0.82
Experiment	0.03	1	0.86
Time	**6.40**	**1**	**0.01**
Treatment × experiment	**9.93**	**2**	**0.01**
Treatment × time	4.83	2	0.09
Experiment × time	2.20	1	0.14
Treatment × experiment × time	4.33	2	0.11

Social cohesion was also higher in the morning (*χ* ^2^ = 6.23, df = 1, *p* = 0.013; Table 1 and Appendix S2, Figure S2 and Table S6), with shoal area being on average 11% smaller and mean neighbor distance 5% shorter in the morning compared to the afternoon. This effect was true across all treatments and therefore likely driven by time of day rather than the visibility level in the flux treatment.

#### 3.1.3 Aggression (Principal Component 3)

Across all three treatments and both experiments there were an average of 5.09 ± 0.55 aggressive contacts, 9.30 ± 0.77 chases, and 3.33 ± 0.24 lateral displays per 10‐min behavior recording. There were no significant differences in fish aggression between experiments (clay and dye) or time of day (morning and afternoon; Table 1). We detected a significant effect of treatment (*χ* ^2^ = 13.55, df = 2, *p* = 0.001; Table 1 and Appendix S2, Figure S3 and Table S6), with fish performing more aggressive behaviors in the clear and flux treatments compared to the stable treatment (post hoc comparison: clear × stable, *t*‐ratio = 2.44, df = 213, *p* = 0.041; post hoc comparison: flux × stable, *t*‐ratio = 3.66, df = 213, *p* = 0.001; Figure 4d and Appendix S2, Table S7), though there was no significant difference between the clear and flux treatments (Appendix S2, Table S7). Fish performed on average 176% more aggressive contacts, 32% more chases, and 30% more lateral displays in the clear treatment compared to the stable treatment, and fish performed on average 228% more aggressive contacts, 119% more chases, and 45% more lateral displays in the flux treatment compared to the stable treatment.

#### 3.1.4 Anxiety (Principal Component 4)

Across all three treatments and both experiments the fish stayed close to the nearest wall, maintaining an average distance of 3.21 ± 0.04 cm from the tank walls. Fish also showed an average maximum speed of 20.10 ± 0.42 cm/s. We detected a significant treatment/experiment interaction on anxiety (*χ* ^2^ = 9.93, df = 2, *p* = 0.007; Table 1 and Appendix S2, Figure S4 and Table S6) but this effect did not survive under post hoc analysis (Figure 4b and Appendix S2, Table S7) and is therefore of low importance. Fish showed more anxiety‐like behaviors in the afternoon compared to the morning (*χ* ^2^ = 6.40, df = 1, *p* = 0.011; Table 1 and Appendix S2, Figure S4 and Table S6) with fish showing an on average 8% higher maximum speed and staying on average 2% closer to the nearest wall in the afternoon compared to the morning.

### 3.2 Optomotor Response Assay

#### 3.2.1 Optomotor Responses

**FIGURE 5:**
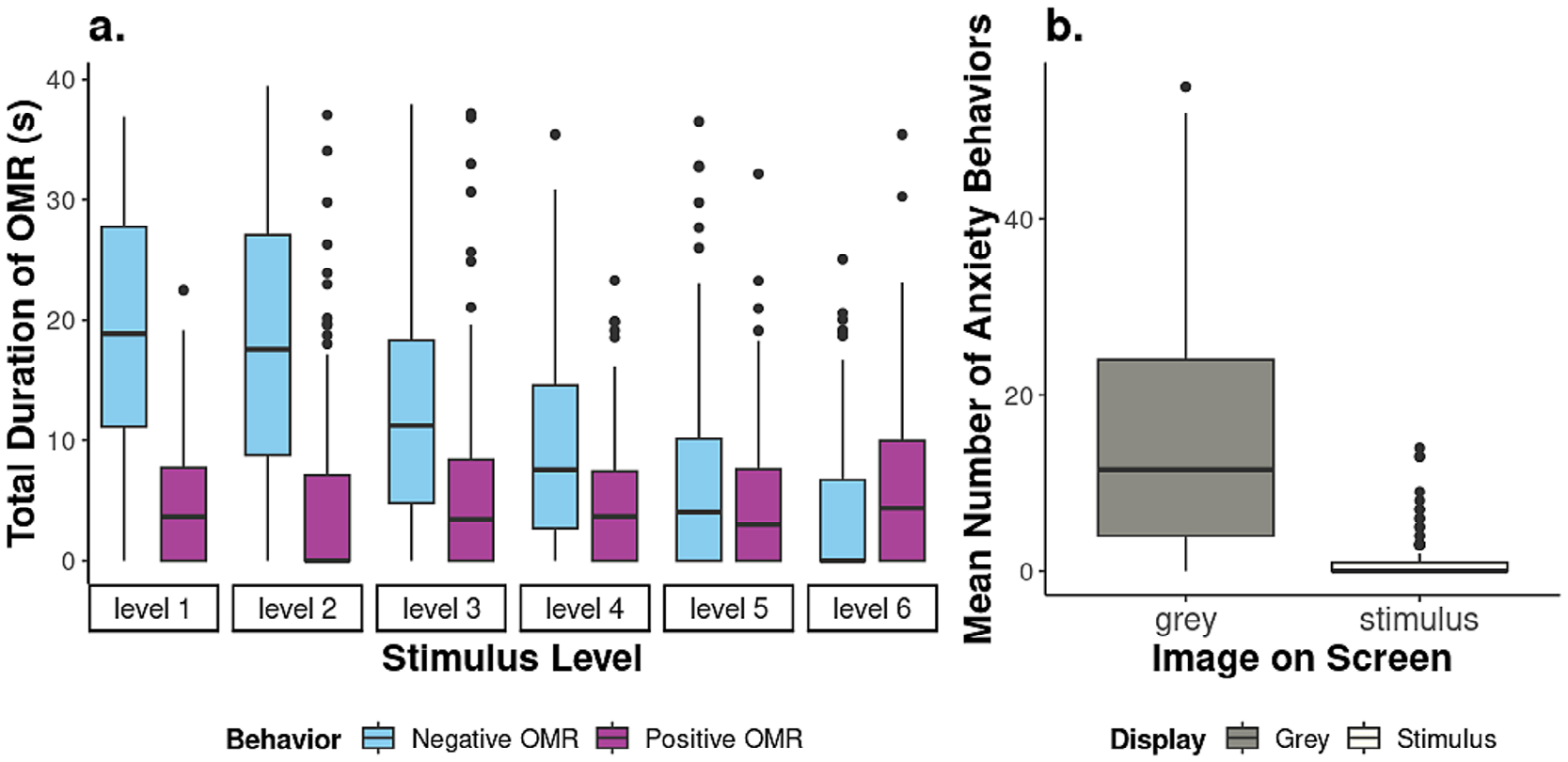
Summary of optomotor response assay behaviors. Box plot formatting is the same as in Figure 4. (a) The positive and negative OMR at each stimulus level of the OMR assay. The negative OMRs are represented with pale blue and the positive OMRs are represented with purple. (b) The anxiety‐like behaviors displayed during the OMR assays. Anxiety‐like behaviors performed during an interstitial gray screen are represented with dark gray while anxiety‐like behaviors performed during a stimulus are represented with off‐white.

There were relatively few positive OMRs while negative OMRs were more common and followed a response pattern more representative of a visual acuity assay, with responses decreasing as the stimulus levels increased and the wedges became more difficult to perceive (i.e., increases in spatial frequency; Figure 5a). As mentioned previously, adult zebrafish are more likely to perform negative OMRs during OMR assays where the visual stimulus is displayed on the bottom of the tank (Bak‐Coleman et al. 2015; Gore et al. 2024), as was the case for our study. We therefore present only the negative OMR results here (please see Appendix S3, Text S1 and Figure S5 for our positive OMR results).

Across all treatments and experiments fish spent about 30% of the trial time performing negative OMRs, with the fish spending an average 70.22 ± 3.10 s performing negative OMRs out of the total 240 s of stimulus presentation. Similarly, fish spent an average of 11.70 ± 0.40 s performing negative OMRs per stimulus level out of a total of 40 s of stimulus presentation.

The fish generally decreased the amount of time they spent performing negative OMRs as the stimulus level increased and the stimuli became more difficult to perceive (Figure 5a and Appendix S3, Table S8 and Figure S6). Surprisingly, the fish spent more time performing negative OMRs in the low visibility OMR assays (both suspended sediments and dye) than in the clear water OMR assays (on average 30% more, *χ* ^2^ = 17.00, df = 1, *p* < 0.0001; Table 2 and Appendix S3, Table S8 and Figure S6). There was no significant effect of treatment exposure (clear, stable, or flux) on negative OMRs (Table 2 and Appendix S3, Table S8). Interestingly, females decreased their rates of negative OMRs 15% faster than males (post hoc comparison: stimulus level × sex, *χ* ^2^ = 4.20, df = 1, *p* = 0.04; Table 2 and Appendix S3, Tables S8 and S9, Figure S6). However, this effect of sex should be interpreted with caution due to its loss during the model sensitivity analysis (Appendix S1, Table S5).

**TABLE 2:** Statistical results for the general linear mixed models of the OMR assays. In the first column, “treatment” refers to the visual treatment condition (clear, stable sediments, stable dye, flux sediments, flux dye), “assay water clarity” refers to the visibility during the OMR assay (clear, low visibility), “log(stimulus level)” refers to the log of the stimulus level (stimulus levels 1–6), and “sex” refers to the sex of the fish (male, female). The Wald *Χ* ^2^ value in the second column is the test statistic. “df” in the third column stands for “degrees of freedom.” The fourth column is the *p*‐value, where bold font indicates *p* < 0.05.

Term	Wald *Χ* ^2^	df	*p*
Positive optomotor response			
Treatment	2.25	4	0.69
Assay water clarity	0.47	1	0.49
Log(stimulus level)	0.30	1	0.58
Sex	**12.28**	**1**	**< 0.001**
Treatment: assay water clarity	4.72	4	0.32
Treatment: log(stimulus level)	2.56	4	0.63
Treatment:sex	**11.39**	**4**	**0.02**
Assay water clarity: log(stimulus level)	**6.39**	**1**	**0.01**
Assay water clarity:sex	2.92	1	0.09
Log(stimulus level):sex	1.79	1	0.18
Negative optomotor response			
Treatment	7.59	4	0.11
Assay water clarity	**17.00**	**1**	**< 0.001**
Log(stimulus level)	**221.75**	**1**	**< 0.001**
Sex	1.99	1	0.16
Treatment: assay water clarity	3.18	4	0.53
Treatment: log(stimulus level)	3.77	4	0.44
Treatment:sex	4.18	4	0.38
Assay water clarity: log(stimulus level)	0.25	1	0.62
Water clarity:sex	0.01	1	0.93
Log(stimulus level):sex	**4.20**	**1**	**0.04**
Anxiety‐like behaviors			
Treatment	4.40	4	0.35
Water clarity	**8.33**	**1**	**0.004**
Image on screen	**146.20**	**1**	**< 0.001**
Sex	1.93	1	0.16
Treatment: water clarity	5.74	4	0.22
Treatment: image on screen	4.86	4	0.30
Treatment:sex	**22.82**	**4**	**< 0.001**
Water clarity: image on screen	1.46	1	0.23
Water clarity:sex	1.11	1	0.29
Image on screen:sex	0.38	1	0.54

#### 3.2.2 Anxiety‐Like Behaviors

Across all treatments and experiments fish performed an average of 16.79 ± 1.33 anxiety‐like behaviors (jumps and agitation behavior) during the 460‐s OMR assay. Fish performed 44% more anxiety‐like behaviors in the clear water OMR assay compared to the low visibility OMR assay (*χ* ^2^ = 8.33, df = 1, *p* = 0.004; Table 2 and Appendix S3, Table S8 and Figure S7). Interestingly, the fish also performed 1108% more anxiety‐like behaviors during the interstitial gray resting screens between stimulus presentations than during stimuli (*χ* ^2^ = 146.20, df = 1, *p* < 0.0001; Table 2, Figure 5b, and Appendix S3, Table S8 and Figure S7). More specifically, fish performed an average of 1.28 ± 0.24 anxiety‐like behaviors during the 240 s of stimulus presentations and an average of 15.51 ± 1.30 anxiety‐like behaviors during the 220 s of interstitial gray screens.

Female fish showed a different anxiety‐like response based on treatment (treatment × sex, *χ* ^2^ = 22.82, df = 4, *p* = 0.0001; Table 2 and Appendix S3, Table S8 and Figure S7), with female zebrafish from the stable dye treatment performing 185% more anxiety‐like behaviors compared to female fish from the flux dye treatment (post hoc comparison: female dye flux × female dye stable, *z*‐ratio = 3.52, *p* = 0.043; Appendix S3, Table S9) and female fish from the clear treatment also performing 209% more anxiety‐like behaviors compared to female fish from flux dye treatment (post hoc comparison: female flux dye × female clear, *z*‐ratio = −2.78, *p* = 0.004; Appendix S3, Table S9). There was no clear effect of the clay treatments on female fish anxiety‐like behaviors, and there was no clear effect of any treatment (dye or clay) on male fish anxiety‐like behavior (Appendix S3, Table S9).

## 4 Discussion

In this study, we tested the effects of suspended sediments and dye on zebrafish behavior and visual perception. Contrary to our predictions that visual pollution would decrease all social interactions, we found that suspended sediments and dye decreased fish aggression while increasing social cohesion. Similarly, while we predicted that visual pollution would decrease fish movement, in reality the rate of movement depended on whether sediments or dye were used to create the poor visible environment. Fish moved more in fluctuating dye conditions but less in stable suspended sediment conditions, though this result was only observed in the morning. General fish anxiety‐like behavior appeared to be minimally affected by a 2‐day exposure to low visibility, but when fish were placed individually into the OMR assay the fish displayed fewer anxiety‐like behaviors under both suspended sediment and dye assay conditions compared to the clear water assays. These results suggest reduced visibility might actually ameliorate anxiety in zebrafish under particularly stressful conditions, such as being placed alone in an unfamiliar testing arena. This might occur either because the lower visibility obscures potential danger or because low visibility conditions offer perceived protection from danger. In contrast to the observed effects of treatments on behavior, we found no significant effects of exposure to sediments or dye on the fish's ability to detect the stimuli in our OMR assay, which could indicate either that treatments did not influence the fish's ability to detect moving stimuli or that our assay design was not sufficiently sensitive for detecting differences in vision under low visibility conditions.

Fish social cohesion and aggression were inversely related, suggesting that zebrafish adaptively increase their shoaling behavior and reduce their aggression when visibility is poor. Shoaling is a common antipredator behavior among fish species (Pitcher 1998) and conspecific aggression can leave participants vulnerable to predation, likely due to distraction (Kim et al. 2004; Meuthen et al. 2016; Wisenden and Sargent 1997). So, the increase in sociability and reduction in aggression when visibility is poor likely represents an adaptation to defend against predators. Many species thought to prey on wild zebrafish (Suriyampola et al. 2015) such as catfish (*Bagarius* spp.), freshwater eels (*Macrognathus* spp.), and snakeheads (*Channa* spp.), are primarily nonvisual hunters (Gisbert et al. 2022; Prasada Rao and Kulkarni 1991) or have eyes that are adapted to turbid water (Bhattacharjya et al. 2018), so predators are likely to remain a threat even in highly turbid waters. It may therefore be evolutionarily advantageous for zebrafish to engage in antipredator behavior any time their visibility is impaired in case obscured predators are nearby. Notably, zebrafish have been shown to primarily identify conspecifics through olfactory rather than visual cues (Santacà et al. 2021), so the observed increase in social proximity may be an antipredator response rather than an attempt to find group mates.

Fluctuation of the visual environment had no clear impact on fish social cohesion but counteracted the increase in fish aggression caused by low visibility. This likely represents different levels of priority for shoaling versus aggression in the face of potential danger, with increased shoaling acting as the primary defense mechanism and reduced aggression only occurring when visibility levels are consistently poor. This is further supported by our earlier research on suspended sediments and dye which showed little to no effect of low visibility on aggression at higher visibility levels (and lower concentrations of clay and dye) than those tested in this study (Anderson, Qiu, et al. 2026; Anderson and Balshine 2026).

Since consistent suspended sediment exposure decreased fish movement relative to clear water while fluctuating dye exposure increased movement relative to clear water, it is possible that zebrafish have different adaptations to turbidity versus low light conditions. Suspended sediments are usually caused by rainfall washing terrestrial sediments into aquatic waterways (Gillain 2005; Loperfido et al. 2010; Navratil et al. 2012). Suspended sediments caused by rainfall are an especially frequent occurrence in the zebrafish's native range (Clift and Jonell 2021; Panda et al. 2011; Suriyampola et al. 2015), and are likely to both cover a relatively broad geographic range and persist for an extended duration (Gillain 2005; Loperfido et al. 2010; Navratil et al. 2012), making such turbid conditions difficult to avoid or swim away from. Zebrafish may have consequently adapted a “wait it out” strategy to reduce movement during prolonged periods of turbidity to minimize the chance of encountering a predator that they cannot see clearly or accidentally straying away from the safety of their shoal. That zebrafish movement levels were lowest under stable turbidity rather than fluctuating turbidity in the current study further supports this hypothesis. While rain‐caused suspended sediments are largely unavoidable, sediments can also be suspended through benthic disturbance such as an animal walking through a stream (Adámek and Maršálek 2013; Kjelland et al. 2015), which would be both a more ephemeral effect and impact a smaller area of the environment. Although the difference in fish movement was only detected in the morning, this is likely driven by zebrafish's overall higher activity levels in the morning (Hurd et al. 1998; Krylov et al. 2021; MacPhail et al. 2009) rather than a specific effect of time of day on antipredator response. We acknowledge that there is relatively little information about suspended sediment levels across the zebrafish native range. However, we argue that the overall trends observed in this study are relevant, particularly as we observed similar differences in movement when fish were exposed to dye and suspended sediment of lower concentrations (Anderson, Qiu, et al. 2026).

The faster movement of the fish under fluctuating dye conditions likely indicates avoidance behavior like that observed in fish under heat stress (McDonnell and Chapman 2015) or hypoxia (Herbert and Steffensen 2006; Petersen and Petersen 1990), where fish will increase their movement in an apparent attempt to swim away to more favorable environmental conditions. Avoidance behavior may be an adaptive response under natural conditions, where fluctuating low light resembles an avoidable environmental condition such as shadows, while constant low light represents less avoidable environmental conditions such as an overcast sky. Alternatively, fluctuating dye conditions may have altered the zebrafish's perception of daytime. Consistently low light lowers the overall amount of light available to the fish while leaving the light cycle itself unchanged. Meanwhile, in our flux dye treatment, the addition of dye produced an extended dark period in the morning followed by a lighter period in the afternoon, which could have led to an extended period of perceived dawn (before the full light of morning), when the zebrafish are most active (Hurd et al. 1998; Krylov et al. 2021; MacPhail et al. 2009). In either case, zebrafish movement was fundamentally different between suspended sediment and dye conditions, casting doubt on manufacturer claims that aquatic dyes are fully ecologically safe for use in habitats with fish or other aquatic organisms (Cuthbert et al. 2024). We note that current information about dye concentrations in the “wild” is limited and therefore the concentrations used in our experiment may not be reflective of concentrations wild fish may encounter. However, considering the increasing use of pond/aquatic dyes for both environmental control and esthetic purposes (Cuthbert et al. 2024; Hussner et al. 2017) and the ease in which the public can acquire pond dyes for personal use, our results highlight the need for further research on the environmental effects of dyes on fish and other aquatic organisms.

Low visibility conditions decreased anxiety‐like behaviors during the OMR assay but did not influence anxiety‐like behavior during the main experimental recordings, suggesting that low visibility conditions may reduce zebrafish stress levels during a high‐stress event. The OMR assay was a stressful event where fish were removed from their experimental tanks and placed alone into an unfamiliar arena. Low visibility conditions during the assay may have made the fish feel safer, as the low visibility reduced their ability to detect potential danger (Sohel and Lindström 2015). Alternatively, the fish may have felt safer because across evolutionary timeframes low visibility conditions provided some kind of advantage to zebrafish. Some recent research suggests that low visibility conditions tend to negatively impact predator detection of prey more than prey detection of predators (Zanghi and Ioannou 2025), which would place a prey species like the zebrafish at a relative advantage during low visibility conditions. This second hypothesis is further supported by the lack of a change in anxiety‐like behavior under low visibility during the general behavioral recordings when fish were in groups. The reduced signs of anxiety afforded by the low visibility conditions was likely only apparent during the OMR assay because it was perceived as a high‐risk situation due to the fish being captured in nets and placed alone in an unfamiliar arena. It is also possible the fish reduced their performance of anxiety‐like behaviors without a corresponding reduction in anxiety, such as by suppressing unnecessary movements like anxiety‐like behaviors when in a low visibility environment to avoid accidentally bumping into an unseen danger.

Experience or exposure to the different visual conditions did not influence how much time the fish spent performing either positive or negative OMRs, and low visibility conditions actually increased the duration fish spent responding to the assay stimuli. Given the relatively short duration of our treatments (4 days) it is perhaps unsurprising that experience with suspended sediments or dye failed to have a strong effect on zebrafish capacity to perceive moving stimuli. While some degree of visual plasticity is common among fish species (Bertinetti and Torres‐Dowdall 2025; Shaughnessy and Cortesi 2024), similar levels of phenotypic visual plasticity are generally thought to occur over time scales on the order of weeks to months rather than days (Luehrmann et al. 2018; Suriyampola et al. 2020). The fact that fish spent more time performing negative OMRs during the low visibility OMR assays than the high visibility assays (with clear water) was likely not driven by sediments or dye increasing the ability of the fish to discriminate moving stimuli, but rather was likely an artifact resulting from the higher number of anxiety‐like behaviors performed during the high visibility assays. Fish were less likely to attend to the moving stimuli while stressed (personal observation), which probably led to fewer OMRs even when the stimuli was visually easier to discriminate. We also wish to emphasize that by presenting our stimuli on a computer monitor the stimuli consisted of emitted light rather than light reflected off an object. This emitted light likely increased the visibility of the stimuli. The response of our zebrafish to the OMR stimuli is therefore unlikely to be fully representative of fish visibility or behavior in other aspects of our experiment, such as interaction with conspecifics or proximity to tank walls. For comparison, a study by Nieman et al. (2018) found that elevated turbidity did interfere with the ability of emerald shiners ( *Notropis atherinoides* ) and walleye ( *Sander vitreus* ) to detect a non‐digital OMR stimulus. We therefore advise caution when using digitally‐presented stimuli under low visibility conditions to avoid artificially inflating the visibility of these stimuli.

However, we do believe that our OMR assay was successful in measuring some degree of visual discrimination of moving stimuli unrelated to treatment conditions. The fish displayed a clear, stereotypical decrease of negative OMRs as the stimulus level in the assay increased and the wedges became more difficult to discern, which is expected in successful OMR assays (Krauss and Neumeyer 2003; Maaswinkel and Li 2003; Suriyampola et al. 2018). In contrast, the fish showed no such decrease in positive OMRs over the course of the assay, indicating the change in behavior was not merely a side effect of decreasing movement rates (habituation) or altered movement patterns over the course of the assay. The fish also showed dramatically fewer anxiety‐like behaviors during stimulus presentations compared to during the interstitial gray screens, suggesting the fish paid attention to the stimuli.

Though zebrafish are typically reported to respond to OMR assays with positive OMRs (Krauss and Neumeyer 2003; Maaswinkel and Li 2003; Suriyampola et al. 2018), our fish primarily responded with negative OMRs, reinforcing results observed by Bak‐Coleman et al. (2015) and Gore et al. (2024) in adult zebrafish. This is particularly notable because while traditional OMR assays display their visual stimuli vertically, either along a flat wall or on the inside surface of a cylinder surrounding the subject (Krauss and Neumeyer 2003; Maaswinkel and Li 2003; Suriyampola et al. 2018), our study and those of Bak‐Coleman et al. (2015) and Gore et al. (2024) displayed the visual stimuli on the floor of the testing tanks. This difference in response behavior may be an adaptation facilitating rheotaxis, enabling the fish to better maintain their position under water flow, though further research is needed to verify this conclusion (Bak‐Coleman et al. 2015).

We acknowledge that the use of a laboratory‐bred stock zebrafish that had been in the lab for generations means that the behavioral responses observed may not be entirely representative of how wild zebrafish would respond under similar conditions. Laboratory‐bred fish are generally reared in simpler, clearer, and more stable environments than their wild counterparts, so it is possible they have lost some of their adaptation to the low visibility and fluctuating environmental conditions found in their home range. It is even possible that wild zebrafish would show greater stress in clear water compared to turbid conditions, as clear water in the wild is both necessarily much less common in the wild than in the laboratory and may leave zebrafish more vulnerable to predation (Zanghi and Ioannou 2025). However, the fact that we observed similar differences in zebrafish movement in response to suspended sediments and dye in this study and in a previous study using weaker concentrations of clay and dye (Anderson, Qiu, et al. 2026) suggests that many of these responses are strongly ingrained. Our research is some of the first to compare the effects of suspended sediments and dye on fish and the first to compare the effects of fluctuating versus consistent visibility on fish behavior. We hope that future research will expand upon our methods under more naturalistic conditions.

## 5 Conclusions and Future Directions

Here, we showed that zebrafish appear to primarily respond to visual pollution by forming tighter shoals and either attempting to avoid/escape polluted areas or by moving more cautiously through them. Zebrafish provide a useful model for adaptive responses to visual pollution as they evolved under frequently turbid conditions (Arunachalam et al. 2013; Bilotta and Saszik 2001; Suriyampola et al. 2015), and we hope our research will be used as a reference to help evaluate the vulnerability of other, more imperiled species to visual pollution. We also found that even seemingly similar forms of visual pollution, specifically suspended sediments and black dye, can have diverging effects on fish behavior. This is particularly notable given the increasing use of aquatic dyes in environmental management (Cuthbert et al. 2024; Hussner et al. 2017) and manufacturer claims that aquatic dyes are safe (Cuthbert et al. 2024). Furthermore, we showed that fluctuating visibility modulates the impact of low visibility conditions on fish behavior, highlighting the need for further research on the impacts of environmental variability on animal behavior more generally. In this study we examined zebrafish in highly artificial laboratory conditions with restricted tank sizes and manual stirring of sediments; however, despite the artificial conditions, our results have already revealed that patterns of fluctuation influence the effects of visual environments on fish behavior and different forms of visual pollution can have different behavioral impacts. Behavior is one of the primary mechanisms animals like fish have for interacting with their surroundings, so we hope that our results will encourage future researchers to further tease apart the importance of these interconnected environmental factors on animal behavior.

## Author Contributions

**Hannah M. Anderson:** conceptualization (lead), data curation (lead), formal analysis (lead), methodology (lead), software (lead), visualization (lead), writing – original draft (lead), writing – review and editing (equal). **Emily Elsasser:** data curation (supporting), investigation (lead), methodology (supporting), writing – review and editing (equal). **James B. Barnett:** methodology (supporting), software (supporting), writing – review and editing (equal). **Sigal Balshine:** conceptualization (supporting), funding acquisition (lead), supervision (lead), writing – review and editing (equal).

## Funding

This work was supported by McMaster University, 20019781; Canadian Network for Research and Innovation in Machining Technology, Natural Sciences and Engineering Research Council of Canada, NSERC‐RGPIN‐2022‐05353.

## Conflicts of Interest

The authors declare no conflicts of interest.

## Supporting information


**Appendix S1:** Additional materials and methods tables.
**Table S1:** Comparison of turbidity meter and turbidity tube measurements.
**Table S2:** Black disc visibility measurements of treatments.
**Table S3:** Principal component weights.
**Table S4:** Proportion of variance accounted for by each principal component.
**Table S5:** Sensitivity analysis model comparisons for the negative optomotor response statistical model.
**Appendix S2:** Additional tables and figures for the standardized behavioral recordings.
**Table S6:** Estimated marginal means and confidence intervals of linear mixed model analyses of principal components.
**Table S7:** Post hoc contrasts of significant results from the linear mixed model analyses of the principal components.
**Figure S1:** Levels of movement (as captured by PC 1) across experiment, treatments, and time of day.
**Figure S2:** Levels of social cohesion (as captured by PC 2) across experiment, treatments, and time of day.
**Figure S3:** Fish aggression (as captured by PC 3) across experiments, treatments, and time of day.
**Figure S4:** Fish anxiety‐like behaviors (as captured by PC 4) across experiments, treatments, and time of day.
**Appendix S3:** Additional results, tables, and figures for the OMR assays.
**Text S1:** Positive optomotor response results.
**Table S8:** Estimated marginal means and confidence intervals of general linear mixed model analyses of optomotor response assays.
**Table S9:** Post hoc contrasts of significant results from the general linear mixed model analyses of the optomotor response assays.
**Figure S5:** Total duration of positive optomotor responses across treatment, assay visibility level, and sex.
**Figure S6:** Total duration of negative optomotor responses across treatment, assay visibility level, and sex.
**Figure S7:** Total number of anxiety‐like responses across treatment, assay visibility level, and sex.
**Appendix S4:** Additional analyses and results for representative behaviors.
**Figure S8:** Notable significant effects of (a) mean speed, (b) proximity to the nearest wall, (c) mean neighbor distance, and (d) aggressive contacts.

## Data Availability

All data are available at https://doi.org/10.5281/zenodo.15376462 and all code is available at https://doi.org/10.5281/zenodo.15375997.
