# Supplementary material for: The Effects of Suspended Sediments, Dye, and Their Variation on Fish Behavior
Source: Ecol Evol. 2026 Aug 13;16(8):e74157. doi: 10.1002/ece3.74157 (PMC13471869; doi:10.1002/ece3.74157)

Appendices for
THE EFFECTS OF SUSPENDED SEDIMENTS, DYE, AND THEIR VARIATION ON FISH BEHAVIOR

Hannah M. Anderson[1a*], Emily Elsasser[1b], James B. Barnett[2c], Sigal Balshine[1d]

[1] Department of Psychology, Neuroscience & Behaviour, McMaster University, Hamilton, Ontario, Canada
[2] School of Natural Sciences, Trinity College Dublin, Dublin, Ireland
[a.] ORCiD: 0000-0002-3620-4641
[b.] ORCiD: 0009-0001-3398-4195
[c.] ORCiD: 0000-0001-9789-4132
[d.] ORCiD: 0000-0003-3671-0517

* Corresponding author:
Hannah M. Anderson
E-mail: anderh3@mcmaster.ca, handerson.scleropages@gmail.com

Table of Contents

APPENDIX 1. ADDITIONAL MATERIALS AND METHODS TABLES 3

Table A1. Comparison of turbidity meter and turbidity tube measurements. 3

Table A2. Black disc visibility measurements of treatments. 4

Table A3. Principal component weights. 5

Table A4. Proportion of variance accounted for by each principal component. 5

Table A5. Sensitivity analysis model comparisons for the negative optomotor response statistical model. 6

APPENDIX 2. ADDITIONAL TABLES AND FIGURES FOR THE STANDARDIZED BEHAVIORAL RECORDINGS..7

Table A6. Estimated marginal means and confidence intervals of linear mixed model analyses of principal components... 7

Table A7. Post hoc contrasts of significant results from the linear mixed model analyses of the principal components. 8

Figure A1. Levels of movement (as captured by PC 1) across experiment, treatments, and time of day.. 9

Figure A2. Levels of social cohesion (as captured by PC 2) across experiment, treatments, and time of day... 10

Figure A3. Fish aggression (as captured by PC 3) across experiments, treatments, and time of day. 11

Figure A4. Fish anxiety-like behaviors (as captured by PC 4) across experiments, treatments, and time of day. 12

APPENDIX 3. ADDITIONAL RESULTS, TABLES, AND FIGURES FOR THE OMR ASSAYS 13

Text A1. Positive optomotor response results. 13

Table A9. Post hoc contrasts of significant results from the general linear mixed model analyses of the optomotor response assays. 16

Figure A5. Total duration of positive optomotor responses across treatment, assay visibility level, and sex. 18

Figure A6. Total duration of negative optomotor responses across treatment, assay visibility level, and sex. 19

APPENDIX 4. ADDITIONAL ANALYSES AND RESULTS FOR REPRESENTATIVE BEHAVIORS 21

Figure A8. Notable significant effects of (a) mean speed, (b) proximity to the nearest wall, (c) mean neighbor distance, and (d) aggressive contacts. 23

APPENDIX 1. ADDITIONAL MATERIALS AND METHODS TABLES

Table A1. Comparison of turbidity meter and turbidity tube measurements.

All visibility measurements were conducted by the same researcher (HMA). The researcher prepared three solutions of each concentration of clay and dye (high concentration clay, moderate concentration clay, high concentration dye, moderate concentration dye) following the same methods as used in the experiments. The researcher then measured the visibility of each solution three times each using both the turbidity meter (in nephelometric turbidity units or NTU) and the turbidity tube (in terms of the maximum depth of water under which the Secchi disc at the bottom of the tube was visible or cm).

treatment	concentration	solution #	turbidity meter (NTU)	turbidity tube (cm)
clay	moderate	1	45.1	20.5
			44.3	20.5
			38	20
		2	42.1	20.5
			41.3	20.5
			45.6	19.5
		3	45.8	21
			47.6	21
			39.8	21.5
	high	1	89.6	12
			83.5	12.5
			101	11.5
		2	85.6	11.5
			90.9	12.5
			83.4	12.5
		3	89.9	12.5
			81.6	12.5
			86.4	12
dye	moderate	1	0.9	35.5
			1.04	37
			1.15	36.5
		2	0.9	36.5
			1.19	36
			1.01	35.5
		3	0.83	35.5
			0.92	38
			1.23	37.5
	high	1	1.09	25
			1.38	20.5
			1.37	22.5
		2	1.35	21
			1.19	19
			1.25	20.5
		3	0.83	21
			0.89	18.5
			0.95	20
clear	clear	1	1.29	NA
			0.97	NA
			1.5	NA
		2	1.55	NA
			1.8	NA
			1.44	NA
		3	1.3	NA
			1.17	NA
			1.32	NA

Table A2. Black disc visibility measurements of treatments.

A long plexiglass tank was filled with one solution from each treatment visibility level (low suspended sediments, high suspended sediments, low pond dye, high pond dye) prepared using experimental methods. Three different observers then measured the minimum distance at which a 2 cm diameter matte black disc attached to a rigid rod, viewed horizontally through the treated water, could no longer be discriminated. The mean measurement of the three observers is provided below, along with the turbidity meter and turbidity tube measurements of the same solutions.

**Low Kaolin Clay Concentration**		
**turbidity meter**	**turbidity tube**	**black disc test**
48.8 NTU	20.5 cm	21 cm
**High Kaolin Clay Concentration**		
**turbidity meter**	**turbidity tube**	**black disc test**
94.3 NTU	13 cm	15.5 cm
**Low Pond Dye Concentration**		
**turbidity meter**	**turbidity tube**	**black disc test**
1.11 NTU	65 cm	34.0 cm
**High Pond Dye Concentration**		
**turbidity meter**	**turbidity tube**	**black disc test**
1 NTU	31 cm	20.5 cm

Table A3. Principal component weights.

Principal components (PCs) 1-4 were used for the final analysis of the standard behavioral recordings in the main manuscript. The principal component cutoff was determined by examining the loadings of each PC and selecting those that best captured the primary research questions while simultaneously maximizing the captured variance of the data (Table A4). Bold indicates the primary weights for PC and the double vertical lines represent the PC cutoff.

variable	PC 1	PC 2	PC 3	PC 4	PC 5	PC 6	PC 7	PC 8	PC 9	PC 10	PC 11	PC 12
mean speed	**0.35**	-0.40	-0.10	0.21	0.07	-0.01	-0.23	0.09	-0.01	**-0.57**	**0.52**	0.01
activity	**0.39**	-0.20	-0.08	-0.16	0.26	0.10	-0.32	-0.05	-0.06	-0.18	**-0.74**	-0.08
maximum speed	0.21	-0.39	-0.31	**0.48**	-0.20	-0.07	0.20	0.39	0.16	**0.44**	-0.17	0.03
polarity	-0.15	0.08	-0.04	0.26	**0.86**	-0.35	0.17	0.10	-0.04	0.01	0.01	0.03
shoal area	0.30	**0.56**	-0.21	0.14	-0.09	0.00	0.05	0.11	0.07	-0.21	-0.09	**0.68**
mean neighbor distance	0.30	**0.55**	-0.23	0.13	-0.04	-0.01	-0.02	0.14	0.04	-0.03	0.08	**-0.71**
wall proximity	0.26	-0.08	0.12	**-0.43**	0.16	0.34	0.66	0.36	0.03	-0.11	0.06	-0.03
space use	**0.38**	0.04	-0.09	-0.28	0.28	0.17	-0.34	-0.11	0.05	0.61	0.37	0.15
aggressive contacts	0.21	0.05	**0.64**	0.29	0.06	0.07	0.00	-0.16	**0.65**	0.01	-0.05	-0.03
chases	0.32	0.04	**0.41**	0.35	-0.05	0.08	0.16	-0.19	**-0.72**	0.14	0.00	0.01
follows	0.29	-0.12	-0.26	-0.14	-0.08	-0.43	0.42	**-0.66**	0.13	-0.01	0.01	-0.03
lateral displays	0.20	0.01	**0.34**	-0.32	-0.17	**-0.72**	-0.14	0.40	-0.07	0.05	0.01	0.02

Table A4. Proportion of variance accounted for by each principal component.

Principal components 1-4 were used for the final analysis of the standard behavioral recordings (see above). The double vertical lines represent the principal component cutoff.

	PC 1	PC 2	PC 3	PC 4	PC 5	PC 6	PC 7	PC 8	PC 9	PC 10	PC 11	PC 12
standard deviation	2.08	1.25	1.18	1.04	0.97	0.89	0.81	0.74	0.53	0.45	0.38	0.23
proportion of variance	0.36	0.13	0.12	0.09	0.08	0.07	0.05	0.05	0.02	0.02	0.01	0.00
cumulative proportion	0.36	0.49	0.61	0.70	0.78	0.84	0.90	0.94	0.97	0.98	1.00	1.00

Table A5. Sensitivity analysis model comparisons for the negative optomotor response statistical model.

Results for the models used to test the effect of alternative fits for the stimulus level factor in the negative optomotor response (OMR) statistical model. Results are similar across models, especially between the primary analysis and the quadratic model, though the higher number of comparisons in the ordered factor model results in the loss of the significant stimulus level by sex interaction. **Bold** indicates $p < 0.05$.

Logarithm-Transformed Stimulus Level (Primary Analysis)			
**term**	**X^2**	**df**	**p-value**
treatment	7.59	4	0.11
**water clarity**	**17.00**	**1**	**<0.0001**
**log(stimulus level)**	**221.75**	**1**	**<0.0001**
sex	1.99	1	0.16
treatment:water clarity	3.18	4	0.53
treatment:log(stimulus level)	3.77	4	0.44
treatment:sex	4.18	4	0.38
water clarity:log(stimulus level)	0.25	1	0.62
water clarity:sex	0.01	1	0.93
**log(stimulus level):sex**	**4.20**	**1**	**0.04**
**Quadratic Stimulus Level Test**			
**term**	**X^2**	**df**	**p-value**
treatment	7.59	4	0.11
**water clarity**	**17.00**	**1**	**<0.0001**
**poly(log(stimulus level))**	**221.75**	**1**	**<0.0001**
sex	1.99	1	0.16
treatment:water clarity	3.18	4	0.53
treatment:poly(log(stimulus level))	3.77	4	0.44
treatment:sex	4.18	4	0.38
water clarity:poly(log(stimulus level))	0.25	1	0.62
water clarity:sex	0.01	1	0.93
**poly(log(stimulus level)):sex**	**4.20**	**1**	**0.04**
**Ordered Factor Stimulus Level Test**			
**term**	**X^2**	**df**	**p-value**
treatment	7.63	4	0.11
**water clarity**	**17.81**	**1**	**>0.0001**
**ordered(stimulus level)**	**247.68**	**5**	**>0.0001**
sex	2.16	1	0.14
treatment:water clarity	3.49	4	0.48
treatment:ordered(stimulus level)	10.36	20	0.96
treatment:sex	4.25	4	0.37
water clarity:ordered(stimulus level)	4.72	5	0.45
water clarity:sex	0.03	1	0.86
ordered(stimulus level):sex	7.37	5	0.19

APPENDIX 2. ADDITIONAL TABLES AND FIGURES FOR THE STANDARDIZED BEHAVIORAL RECORDINGS

Table A6. Estimated marginal means and confidence intervals of linear mixed model analyses of principal components.

Estimated marginal means (EMM) were calculated for all main effects of the PC linear mixed models except for the significant interactions, where the EMMs reflect the *post hoc* pairwise comparisons (Table A7).

**PC 1, Movement**							
**time**	**experiment**	**treatment**	**EMM**	**SE**	**df**	**lower CL**	**upper CL**
morning	clay	clear	-0.96	0.39	213	-1.72	-0.20
		flux	-1.42	0.37	213	-2.14	-0.70
		stable	-2.29	0.37	213	-3.01	-1.57
	dye	clear	0.47	0.37	213	-0.25	1.19
		flux	2.29	0.40	213	1.51	3.07
		stable	1.46	0.38	213	0.71	2.22
afternoon	clay	clear	-1.45	0.39	213	-2.21	-0.69
		flux	-0.78	0.37	213	-1.50	-0.06
		stable	-1.52	0.37	213	-2.24	-0.80
	dye	clear	1.02	0.37	213	0.29	1.75
		flux	1.93	0.37	213	1.21	2.65
		stable	1.93	0.37	213	1.20	2.66
**PC 2, Social Cohesion**							
**treatment**			**EMM**	**SE**	**df**	**lower CL**	**upper CL**
clear			0.68	0.19	213	0.30	1.06
flux			-0.31	0.19	213	-0.68	0.07
stable			-0.46	0.19	213	-0.83	-0.08
**experiment**			**EMM**	**SE**	**df**	**lower CL**	**upper CL**
clay			-0.02	0.16	213	-0.33	0.29
dye			-0.04	0.16	213	-0.35	0.27
**time**			**EMM**	**SE**	**df**	**lower CL**	**upper CL**
morning			-0.18	0.13	213	-0.42	0.07
afternoon			0.12	0.12	213	-0.12	0.36
**PC 3, Aggression**							
**treatment**			**EMM**	**SE**	**df**	**lower CL**	**upper CL**
clear			0.09	0.17	213	-0.25	0.43
flux			0.38	0.17	213	0.04	0.72
stable			-0.51	0.17	213	-0.84	-0.17
**experiment**			**EMM**	**SE**	**df**	**lower CL**	**upper CL**
clay			0.15	0.14	213	-0.13	0.42
dye			-0.17	0.14	213	-0.45	0.11
**time**			**EMM**	**SE**	**df**	**lower CL**	**upper CL**
morning			0.09	0.12	213	-0.15	0.32
afternoon			-0.11	0.12	213	-0.34	0.12
**PC 4, Anxiety**							
**experiment**		**treatment**	**EMM**	**SE**	**df**	**lower CL**	**upper CL**
clay		clear	0.23	0.23	213	-0.22	0.67
		flux	-0.44	0.21	213	-0.86	-0.01
		stable	0.19	0.21	213	-0.24	0.61
dye		clear	-0.29	0.22	213	-0.72	0.13
		flux	0.39	0.22	213	-0.05	0.83
		stable	-0.03	0.22	213	-0.46	0.40
**time**			**EMM**	**SE**	**df**	**lower CL**	**upper CL**
morning			-0.13	0.11	213	-0.33	0.08
afternoon			0.14	0.10	213	-0.06	0.34

Table A7. Post hoc contrasts of significant results from the linear mixed model analyses of the principal components.

Post hoc pairwise comparisons were conducted for all significant results with a factor level greater than two. For the significant interactions, pairwise comparisons were conducted on the predictor value of primary interest (treatment) across levels of the interacting variable(s). In the case of overlapping significance (for example, if the main effect of treatment and the interaction between treatment and time were both significant) then only the highest order interaction was analyzed. *Italics* indicates 0.10 < p < 0.5 and bold indicates p < 0.05.

**PC 1, Movement**							
**time**	**experiment**	**treatment**	**estimate**	**SE**	**df**	**t-ratio**	**p-value**
morning	clay	clear - flux	0.46	0.53	213	0.87	0.66
		**clear - stable**	**1.33**	**0.53**	**213**	**2.50**	**0.04**
		flux - stable	0.86	0.52	213	1.67	0.22
	dye	**clear - flux**	**-1.82**	**0.54**	**213**	**-3.38**	**0.002**
		clear - stable	-0.99	0.53	213	-1.87	0.15
		flux - stable	0.83	0.55	213	1.51	0.29
afternoon	clay	clear - flux	-0.67	0.53	213	-1.26	0.42
		clear - stable	0.07	0.53	213	0.13	0.99
		flux - stable	0.74	0.52	213	1.43	0.33
	dye	clear - flux	-0.90	0.52	213	-1.74	0.19
		clear - stable	-0.91	0.52	213	-1.73	0.20
		flux - stable	0.00	0.52	213	-0.01	1.00
**PC 2, Affiliation**							
**treatment**			**estimate**	**SE**	**df**	**t-ratio**	**p-value**
**clear - flux**			**0.99**	**0.27**	**213**	**3.63**	**0.001**
**clear - stable**			**1.14**	**0.27**	**213**	**4.19**	**<0.001**
flux - stable			0.15	0.27	213	0.56	0.84
**PC 3, Aggression**							
**treatment**			**estimate**	**SE**	**df**	**t-ratio**	**p-value**
clear - flux			-0.29	0.24	213	-1.19	0.46
**clear - stable**			**0.60**	**0.24**	**213**	**2.44**	**0.04**
**flux - stable**			**0.89**	**0.24**	**213**	**3.66**	**<0.001**
**PC 4, Boldness**							
**experiment**	**treatment**		**estimate**	**SE**	**df**	**t-ratio**	**p-value**
clay	*clear - flux*		*0.66*	*0.31*	*213*	*2.12*	*0.09*
	clear - stable		0.04	0.31	213	0.13	0.99
	flux - stable		-0.62	0.30	213	-2.04	0.10
dye	*clear - flux*		*-0.68*	*0.31*	*213*	*-2.20*	*0.07*
	clear - stable		-0.26	0.31	213	-0.85	0.67
	flux - stable		0.42	0.31	213	1.34	0.37

Figure A1. Levels of movement (as captured by PC 1) across experiment, treatments, and time of day.

Increasing values of PC 1 are indicative of increasing levels of movement. Experiment 1 (clay) is represented by shades of tan and Experiment 2 (dye) is represented by shades of grey. The treatments are represented by the shades of each color, with the lightest shade representing the clear treatment, the middle shade representing the flux treatment, and the darkest shade representing the stable treatment. The middle line of each box plot represents the median, the lower and upper hinges respectively represent the first and third inter-quartiles, and the upper and lower whiskers respectively extend to the largest and smallest datapoint within 1.5x the interquartile range of the corresponding hinge. Figure A1a shows the data from the morning recordings and Figure A1b shows the data from the afternoon recording. In the morning, the level of movement appears to increase in the clay experiment from the clear condition to the flux condition to the stable condition while in the dye experiment the level of movement appears to be greater in both the flux and stable conditions compared to the clear condition. In the afternoon the movement levels in the clay experiment seem lower than the dye experiment. The movement levels seem similar between treatments for both experiments.

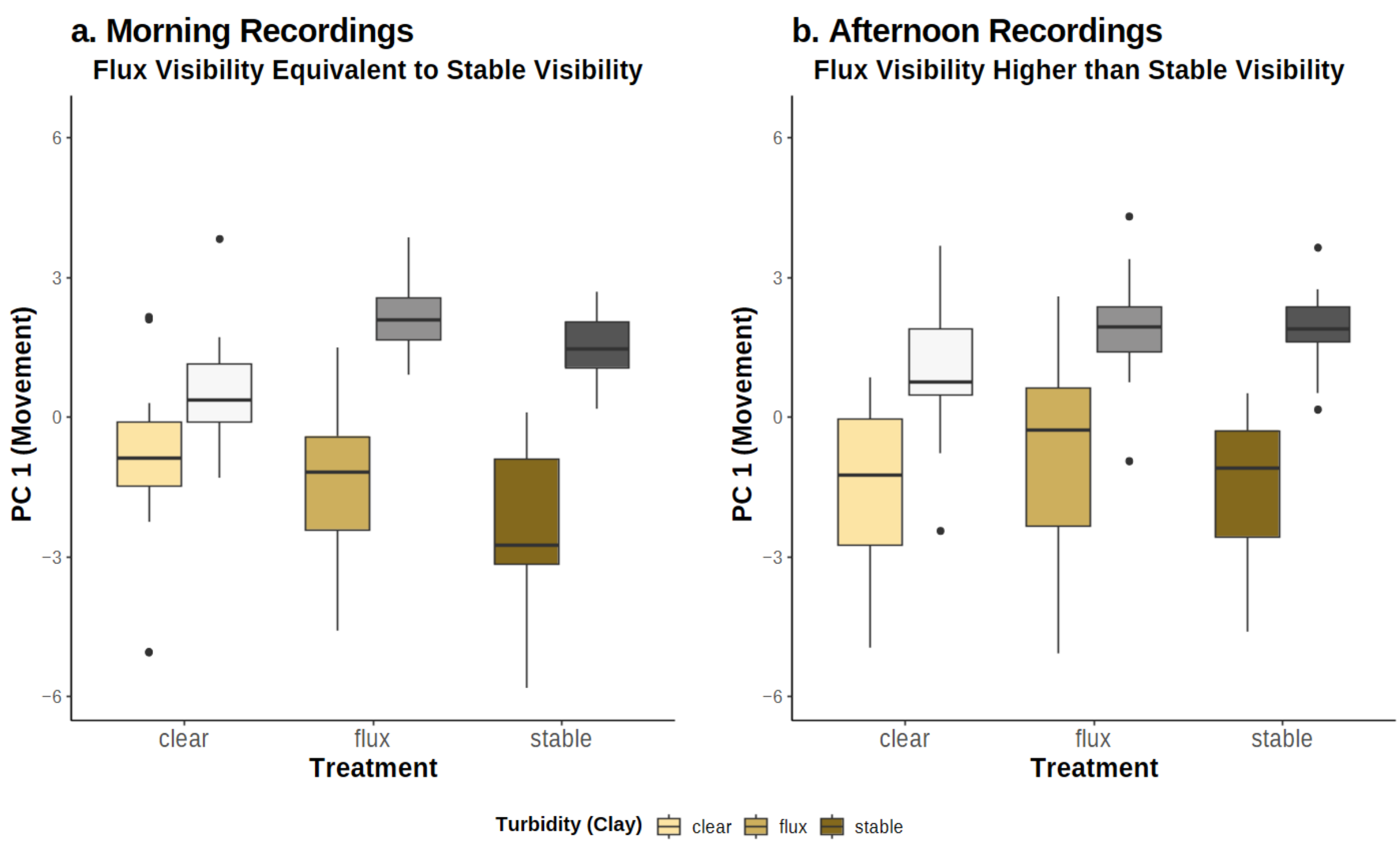

Figure A2. Levels of social cohesion (as captured by PC 2) across experiment, treatments, and time of day.

Social cohesion here is presented as -1*PC 2 so that increasing values on the y-axis represent increasing values of social cohesion. Box plot formatting is the same as in Figure A1. Figure A2a shows the data from the morning recordings and Figure A2b shows the data from the afternoon recording. Both the morning and afternoon recordings show similar patterns in social cohesion, with the stable and the flux conditions showing similar levels of cohesion between them and greater levels of social cohesion compared to the clear condition.

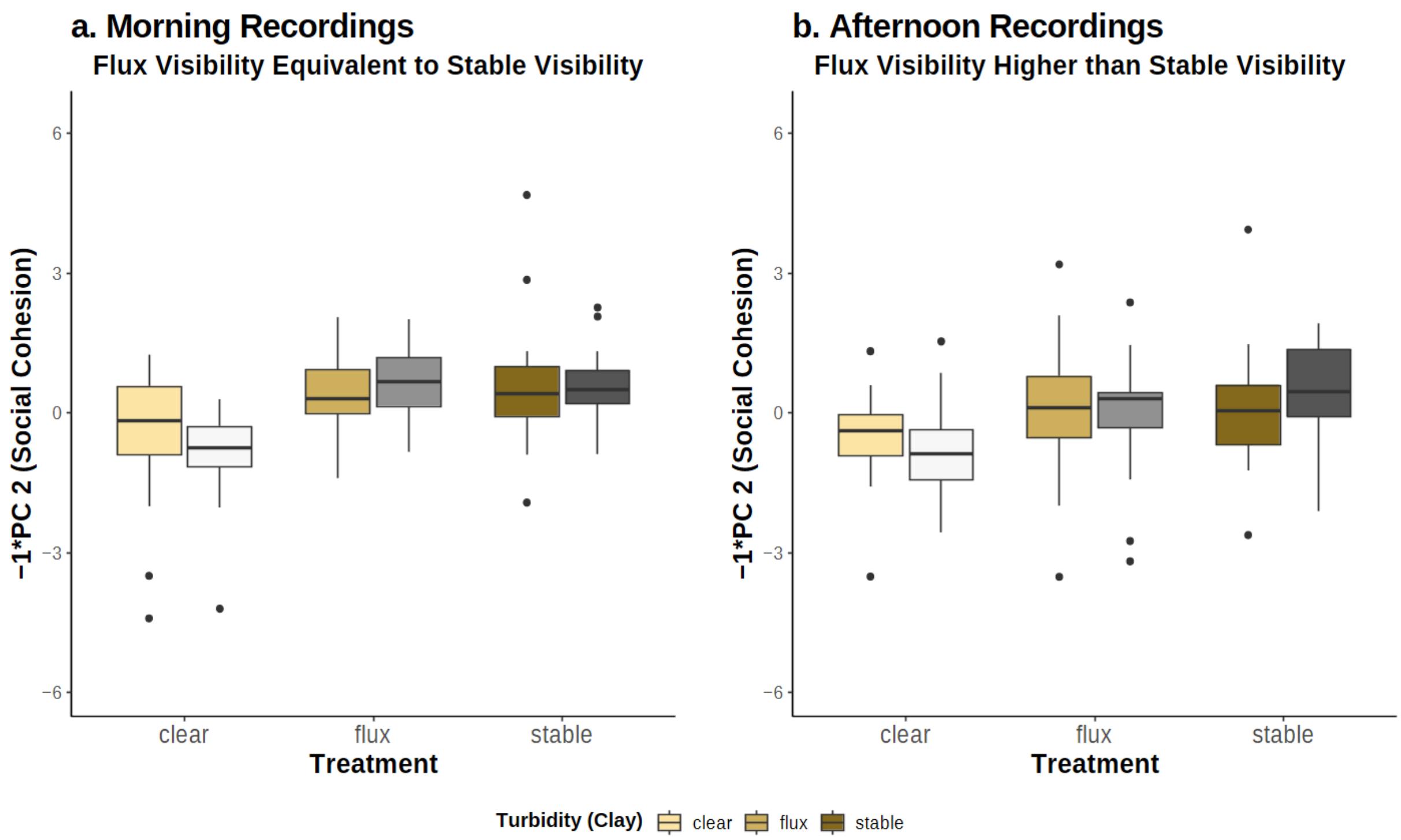

Figure A3. Fish aggression (as captured by PC 3) across experiments, treatments, and time of day.

Increasing values of PC 3 are indicative of increasing levels of movement. Box plot formatting is the same as in Figure A1. Figure A3a shows the data from the morning recordings and Figure A3b shows the data from the afternoon recording. Both the morning and afternoon recordings show similar patterns in aggression, with the clear and flux conditions showing similar levels of aggression between them and the stable condition showing less aggression compared to both the clear and flux conditions.

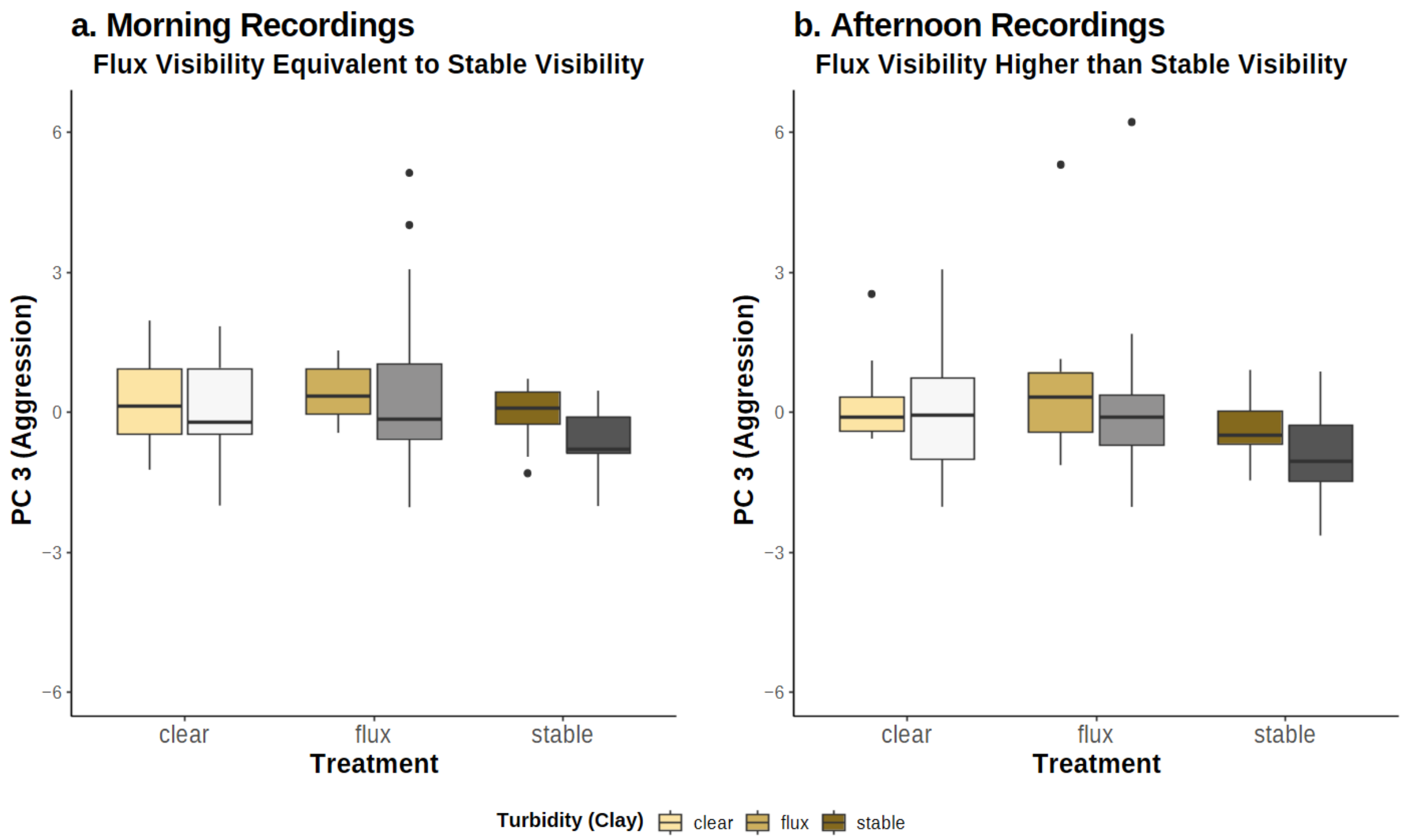

Figure A4. Fish anxiety-like behaviors (as captured by PC 4) across experiments, treatments, and time of day.

Increasing values of PC 4 are indicative of increasing levels of movement. Box plot formatting is the same as in Figure A1. Figure A4a shows the data from the morning recordings and Figure A4b shows the data from the afternoon recording. Anxiety-like behaviors appear to be greater in the morning compared to the afternoon. Anxiety-like behaviors appear to be similar across treatments except for the dye experiment clear condition and the clay experiment flux condition, which show similar levels of anxiety-like behaviors between them and slightly lower levels of anxiety-like behaviors compared to all other conditions.

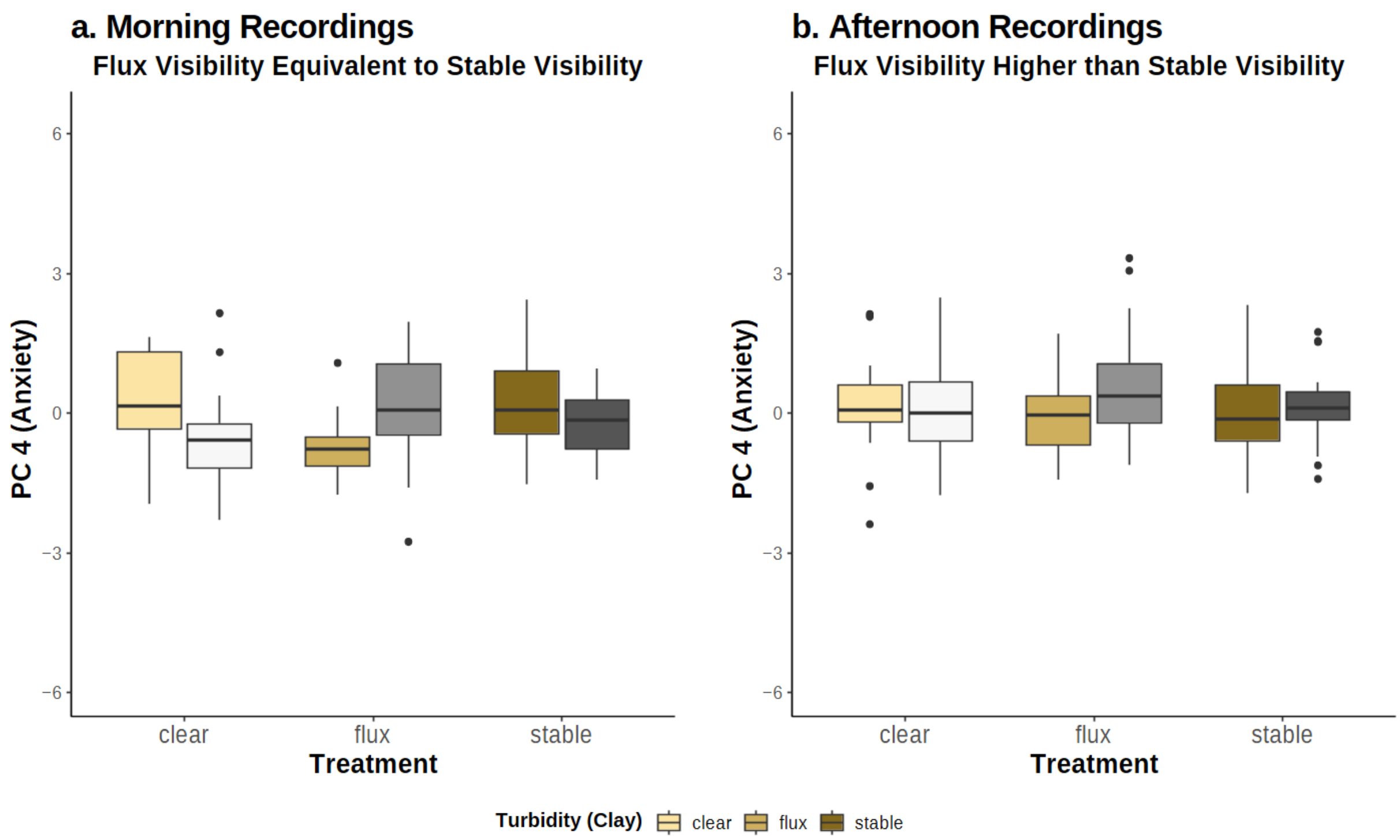

APPENDIX 3. ADDITIONAL RESULTS, TABLES, AND FIGURES FOR THE OMR ASSAYS

Text A1. Positive optomotor response results.

Across all treatments and experiments fish collectively spent an average of 31.59 ± 2.16 seconds per OMR assay performing positive OMRs out of the total 240 seconds of stimulus presentation. Similarly, across all treatments and experiments fish spent an average of 5.26 ± 0.25 seconds per stimulus level performing positive OMRs out of the total 40 seconds of stimulus presentation. In clear water, the fish spent less time performing positive OMRs as the stimulus level increased and became harder to perceive while in low visibility water the trend was the opposite, with the fish spending more time performing positive OMRs as the stimulus level increased ($\chi2 = 6.39$, DF = 1, $p = 0.01$; Tables 2, A8, and A9 and Figures 3 and A5). In the clear water OMR assays, the fish spent on average 1.22x more time performing positive OMRs during the first stimulus level compared to the last stimulus level, while in the low visibility assays fish spent on average 1.90x less time performing positive OMRs during the first stimulus level compared to the last stimulus level. Although we also detected a significant treatment/sex interaction ($\chi2 = 11.39$, DF = 4, $p = 0.02$) this effect did not survive under post hoc analysis (Table 2).

Table A8. Estimated marginal means and confidence intervals of general linear mixed model analyses of optomotor response assays.

Estimated marginal means (EMM) were calculated for all main effects of the OMR assay general linear mixed models except for the significant interactions, where the EMMs reflect the *post hoc* pairwise comparisons (Table A9).

**Positive Optomotor Response**						
**trend**	**assay water clarity**	**EM trend**	**SE**	**Df**	**asymp LCL**	**asymp UCL**
log(stimulus level)	clear	-0.12	0.10	Inf	-0.32	0.08
	low visibility	0.25	0.11	Inf	0.04	0.46
**sex**	**treatment**	**EMM**	**SE**	**Df**	**asymp LCL**	**asymp UCL**
female	clear	1.21	0.14	Inf	0.93	1.49
	clay flux	1.77	0.17	Inf	1.43	2.10
	dye flux	1.46	0.18	Inf	1.11	1.82
	clay stable	1.71	0.17	Inf	1.38	2.04
	dye stable	1.32	0.19	Inf	0.95	1.69
male	clear	1.81	0.12	Inf	1.58	2.04
	clay flux	1.73	0.17	Inf	1.39	2.06
	dye flux	1.78	0.17	Inf	1.45	2.11
	clay stable	1.56	0.18	Inf	1.21	1.90
	dye stable	1.82	0.16	Inf	1.50	2.13
**Negative Optomotor Response**						
**trend**	**sex**	**EM trend**	**SE**	**Df**	**asymp LCL**	**asymp UCL**
log(stimulus level)	female	-0.79	0.07	Inf	-0.93	-0.66
	male	-0.60	0.07	Inf	-0.73	-0.47
**assay water clarity**		**EMM**	**SE**	**Df**	**asymp LCL**	**asymp UCL**
clear		2.10	0.06	Inf	1.98	2.22
low visibility		2.37	0.05	Inf	2.26	2.48
**treatment**		**EMM**	**SE**	**DF**	**asymp LCL**	**asymp UCL**
clear		2.17	0.07	Inf	2.02	2.31
clay flux		2.29	0.10	Inf	2.10	2.49
dye flux		2.40	0.09	Inf	2.21	2.58
clay stable		2.16	0.10	Inf	1.96	2.35
dye stable		2.16	0.10	Inf	1.96	2.36

Assay Anxiety-Like Behaviors						
**sex**	**treatment**	**EMM**	**SE**	**DF**	**asymp LCL**	**asymp UCL**
female	clear	1.80	0.21	Inf	1.40	2.20
	clay flux	1.42	0.30	Inf	0.83	2.02
	dye flux	0.15	0.45	Inf	-0.73	1.03
	clay stable	1.06	0.33	Inf	0.41	1.72
	dye stable	1.56	0.29	Inf	1.00	2.12
male	clear	1.33	0.23	Inf	0.88	1.78
	clay flux	1.98	0.26	Inf	1.47	2.49
	dye flux	1.26	0.36	Inf	0.55	1.97
	clay stable	2.14	0.25	Inf	1.65	2.64
	dye stable	1.77	0.28	Inf	1.22	2.31
**assay water clarity**		**EMM**	**SE**	**DF**	**asymp LCL**	**asymp UCL**
clear		1.59	0.15	Inf	1.29	1.89
low visibility		1.31	0.17	Inf	0.98	1.63
**image on screen**		**EMM**	**SE**	**DF**	**asymp LCL**	**asymp UCL**
gray		2.51	0.11	Inf	2.30	2.72
stimulus		0.39	0.21	Inf	-0.01	0.79

Table A9. Post hoc contrasts of significant results from the general linear mixed model analyses of the optomotor response assays.

Post hoc pairwise comparisons were conducted for all significant results with a factor level greater than two. For the significant interactions, pairwise comparisons were conducted on the predictor value of primary interest (treatment) across levels of the interacting variable(s). In the case of overlapping significance (for example, if the main effect of treatment and the interaction between treatment and water clarity were both significant) then only the highest order interaction was analyzed.

**Positive Optomotor Response**						
**sex**	**treatment**	**estimate**	**SE**	**df**	**z-ratio**	**p-value**
female	clear – clay flux	-0.56	0.22	Inf	-2.52	0.09
	clear – dye flux	-0.25	0.23	Inf	-1.10	0.81
	clear – clay stable	-0.50	0.22	Inf	-2.26	0.16
	clear – dye stable	-0.11	0.23	Inf	-0.47	0.99
	clay flux – dye flux	0.31	0.25	Inf	1.24	0.73
	clay flux – clay stable	0.06	0.24	Inf	0.25	1.00
	clay flux – dye stable	0.45	0.25	Inf	1.77	0.39
	dye flux – clay stable	-0.24	0.25	Inf	-1.00	0.86
	dye flux – dye stable	0.14	0.26	Inf	0.54	0.98
	clay stable – dye stable	0.38	0.25	Inf	1.53	0.54
male	clear – clay flux	0.09	0.21	Inf	0.42	0.99
	clear – dye flux	0.03	0.20	Inf	0.17	1.00
	clear – clay stable	0.26	0.21	Inf	1.22	0.74
	clear – dye stable	0.00	0.20	Inf	-0.02	1.00
	clay flux – dye flux	-0.05	0.24	Inf	-0.22	1.00
	clay flux – clay stable	0.17	0.24	Inf	0.70	0.96
	clay flux – dye stable	-0.09	0.23	Inf	-0.38	1.00
	dye flux – clay stable	0.22	0.24	Inf	0.93	0.89
	dye flux – dye stable	-0.04	0.23	Inf	-0.17	1.00
	clay stable – dye stable	-0.26	0.24	Inf	-1.10	0.81
**slope**	**assay water clarity**	**estimate**	**SE**	**df**	**z-ratio**	**p-value**
stimulus level	**clear – low visibility**	**-0.37**	**0.15**	**Inf**	**-2.53**	**0.01**
**Negative Optomotor Response**						
**slope**	**sex**	**estimate**	**SE**	**df**	**z-ratio**	**p-value**
**stimulus level**	**female - male**	**-0.19**	**0.09**	**Inf**	**-2.05**	**0.04**
**Assay Anxiety-Like Behaviors**						
**sex**	**treatment**	**estimate**	**SE**	**df**	**z-ratio**	**p-value**
female	clear – clay flux	0.38	0.35	Inf	1.10	0.81
	**clear – dye flux**	**1.65**	**0.47**	**Inf**	**3.52**	**0.004**
	clear – clay stable	0.74	0.37	Inf	2.00	0.27
	clear – dye stable	0.24	0.33	Inf	0.73	0.95
	clay flux – dye flux	1.27	0.51	Inf	2.47	0.10
	clay flux – clay stable	0.36	0.43	Inf	0.84	0.92
	clay flux – dye stable	-0.14	0.40	Inf	-0.35	1.00
	dye flux – clay stable	-0.91	0.53	Inf	-1.72	0.42
	**dye flux – dye stable**	**-1.41**	**0.51**	**Inf**	**-2.78**	**0.04**
	clay stable – dye stable	-0.50	0.42	Inf	-1.19	0.76

male	clear – clay flux	-0.65	0.32	Inf	-2.02	0.26
	clear – dye flux	0.07	0.41	Inf	0.17	1.00
	clear – clay stable	-0.81	0.32	Inf	-2.55	0.08
	clear – dye stable	-0.44	0.34	Inf	-1.29	0.70
	clay flux – dye flux	0.72	0.43	Inf	1.66	0.46
	clay flux – clay stable	-0.16	0.34	Inf	-0.47	0.99
	clay flux – dye stable	0.21	0.36	Inf	0.59	0.98
	dye flux – clay stable	-0.88	0.43	Inf	-2.05	0.24
	dye flux – dye stable	-0.51	0.44	Inf	-1.14	0.78
	clay stable – dye stable	0.37	0.36	Inf	1.04	0.84

Figure A5. Total duration of positive optomotor responses across treatment, assay visibility level, and sex.

The clear treatment is represented by the solid blue lines. The clay flux and dye flux treatments are represented by the dashed tan and grey lines respectively. The clay stable and dye stable treatments are represented by the dashed and dotted brown and dark grey lines respectively. Figure A5a represents the positive OMRs displayed by male fish in the clear water OMR assays. Figure A5b represents the positive OMRs displayed by female fish in the clear water OMR assays. Figure A5c represents the positive OMR displayed by male fish in the low visibility OMR assays. Figure A5d represents the positive OMRs displayed by the female fish in the low visibility OMR assays. Across both sexes, assay visibility levels, and all stimulus levels positive OMR duration remains relatively low and consistent.

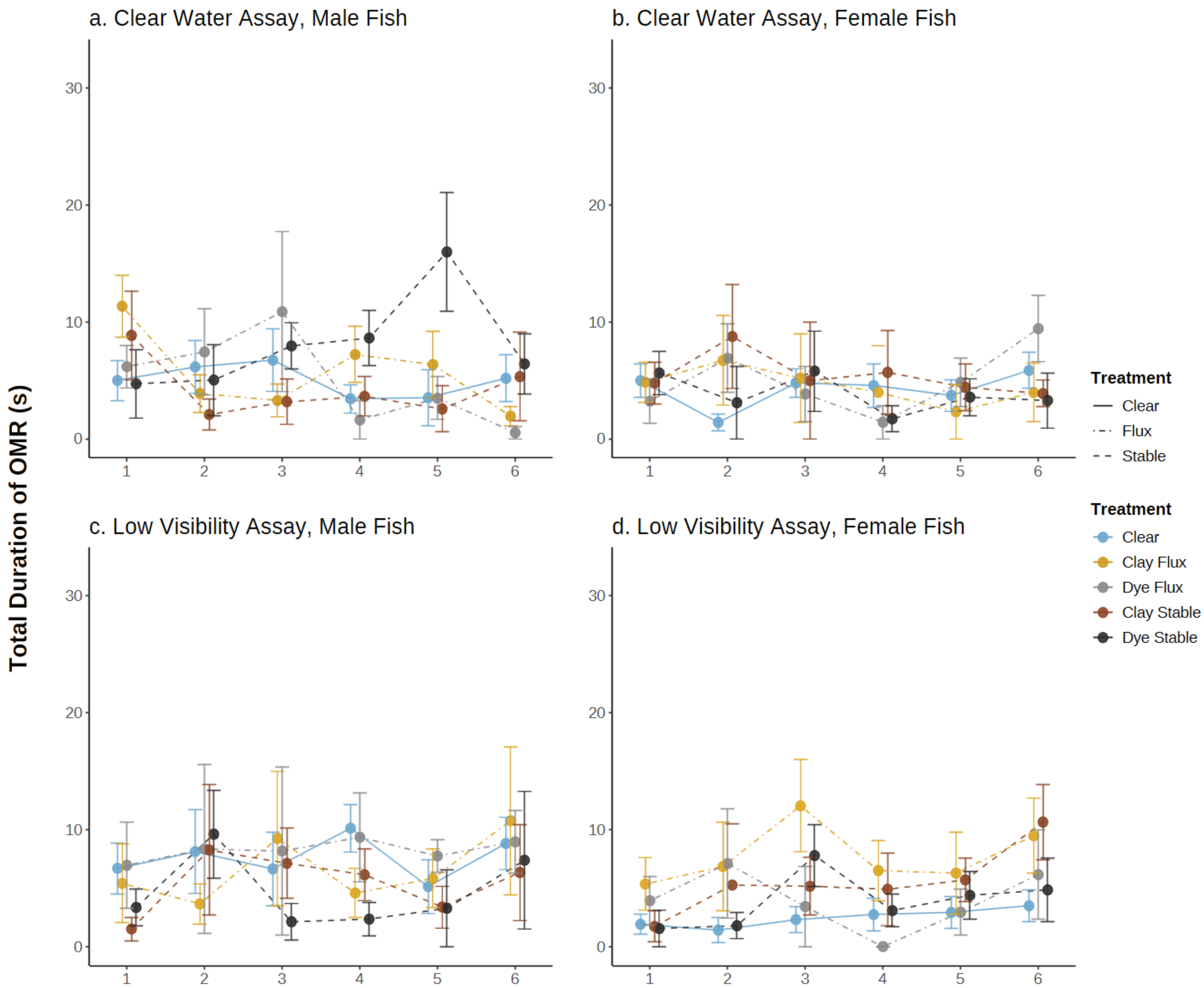

Figure A6. Total duration of negative optomotor responses across treatment, assay visibility level, and sex.

The clear treatment is represented by the solid blue lines. The clay flux and dye flux treatments are represented by the dashed tan and grey lines respectively. The clay stable and dye stable treatments are represented by the dashed and dotted brown and dark grey lines respectively. Figure A6a represents the negative OMRs displayed by male fish in the clear water OMR assays. Figure A6b represents the negative OMRs displayed by female fish in the clear water OMR assays. Figure A6c represents the negative OMR displayed by male fish in the low visibility OMR assays. Figure A6d represents the negative OMRs displayed by the female fish in the low visibility OMR assays. Across both sexes, assay visibilities levels, negative OMR duration starts relatively high at the first stimulus level and decreases steadily with each stimulus level. Female fish appear to start with a longer duration of negative OMRs at the first stimulus level and then decrease to the same duration of OMRs as the male fish by the final stimulus level.

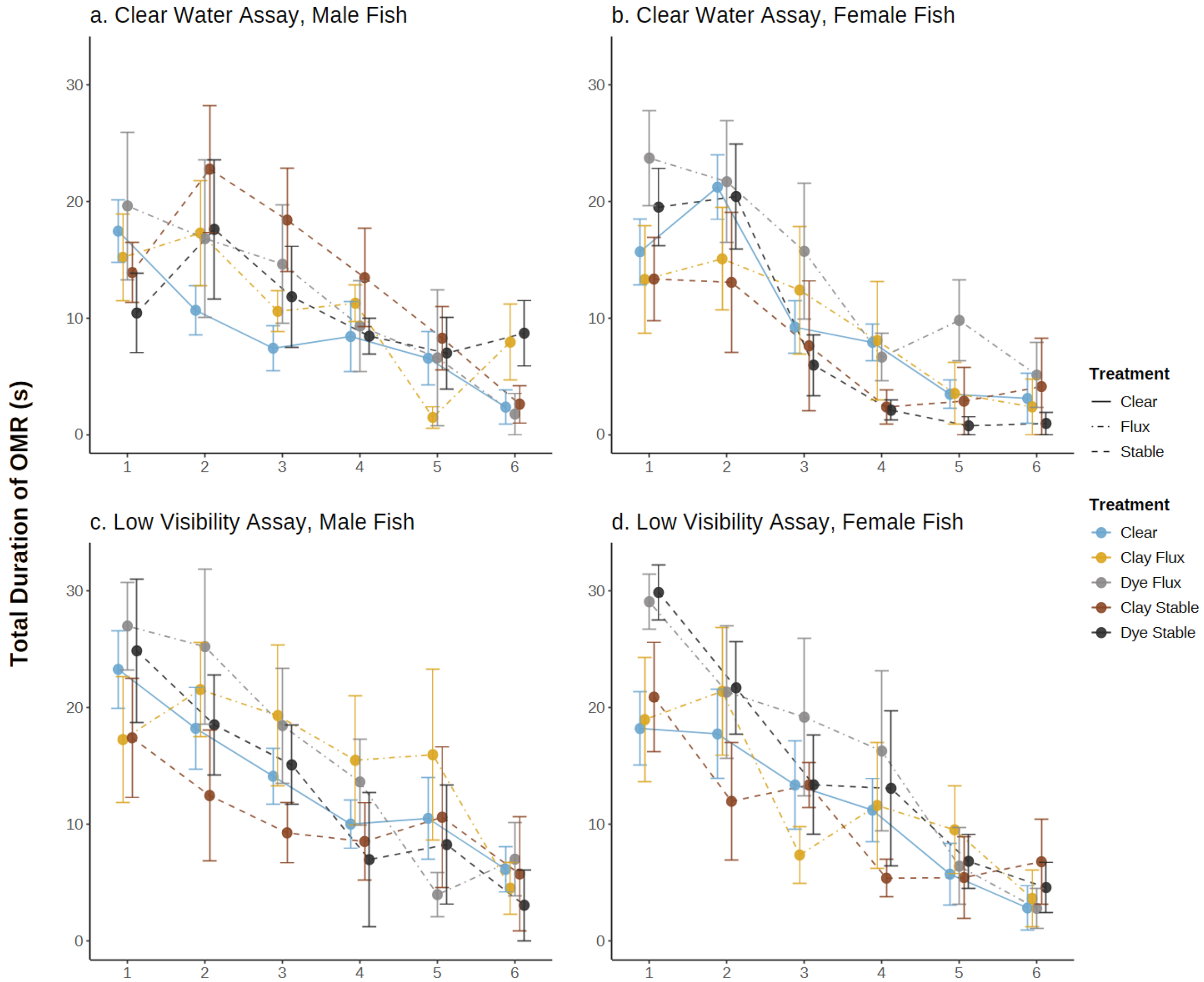

Figure A7. Total number of anxiety-like responses across treatment, assay visibility level, and sex. The clear treatment is represented by blue, the clay flux treatment by tan, the dye flux treatment by light grey, the clay stable treatment by brown, and the dye stable treatment by dark grey. The middle line of each box plot represents the median, the lower and upper hinges respectively represent the first and third inter-quartiles, and the upper and lower whiskers respectively extend to the largest and smallest datapoint within 1.5x the interquartile range of the corresponding hinge. Figure A7a represents the negative OMRs displayed by male fish in the clear water OMR assays. Figure A7b represents the negative OMRs displayed by female fish in the clear water OMR assays. Figure A7c represents the negative OMR displayed by male fish in the low visibility OMR assays. Figure A7d represents the negative OMRs displayed by the female fish in the low visibility OMR assays. Across both sexes and assay visibility levels fish display dramatically more anxiety-like behaviors during the grey interstitial screen than during stimuli, with the number of anxiety-like behaviors performed during stimuli near zero. Across both sexes fish also display more anxiety-like behaviors during the clear water than low visibility assays.

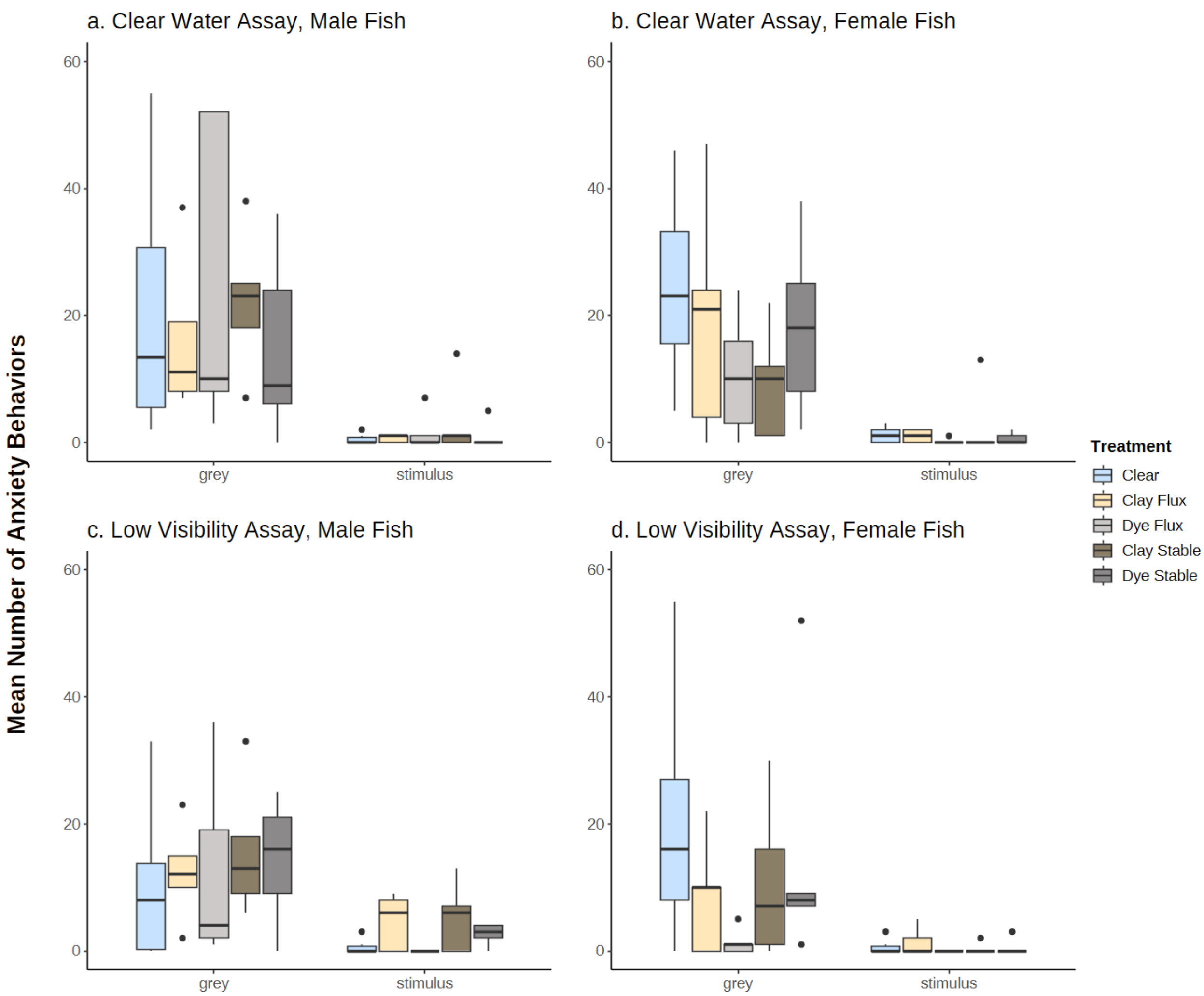

APPENDIX 4. ADDITIONAL ANALYSES AND RESULTS FOR REPRESENTATIVE BEHAVIORS

We used principal component analyses when analyzing our behavioral recordings to reduce the number of analyses and redundancy of highly correlated behaviors. However, a PCA can make the results harder to interpret due to each principal component being made up of multiple behaviors. To provide further clarity, we include here four additional analyses of representative behaviors from each of our four principal components (PC 1, mean speed; PC 2, mean neighbor distance; PC 3, aggressive contacts; and PC 4, proximity to the nearest wall). We fit mean speed, mean neighbor distance, and proximity to the nearest wall with linear mixed models. We included treatment (clear, stable, and flux), experiment (clay and dye), time of day (morning and afternoon), and all of their interactions as fixed effects and shoal ID as a random effect to account for repeated measures. Because aggressive contacts were count data, we instead fit this behavior with a generalized linear mixed model using a Poisson distribution but included the same fixed and random factors as the previous models.

For fish mean speed, we detected a three-way interaction between all three factors in our model (overall model: treatment x experiment x time of day, $\chi^2 = 7.11$, df = 2, $p = 0.03$). In the morning, fish in the dye experiment swam at a higher mean speed in the flux and stable treatments compared to the clear control (*post hoc* pairwise comparisons: dye flux x clear, t-ratio = -3.85, df = 213, $p < 0.001$; dye stable x clear, t-ratio = -3.25, df = 213, $p = 0.004$; Figure A8a). In the afternoon, fish in the dye experiment also swam faster in the stable treatment compared to the clear control (*post hoc* pairwise comparison: dye stable x clear, t-ratio = -2.81, df = 213, $p = 0.01$). We found a similar effect on fish movement in our main manuscript (PC 1) where, in the morning, fish in the stable clay treatment moved less compared to the clear condition and fish in the flux dye treatment moved more compared to the clear condition. Overall, these results still support our findings that there are differences in fish movement between clay and dye conditions.

For mean neighbor distance, we detected a significant effect of treatment ($\chi^2 = 6.55$, df = 2, $p = 0.04$). The *post hoc* analysis revealed that there was a non-significant trend of fish being closer to their neighbors in the stable treatment compared to the clear control (t-ratio = 2.32, df = 213, $p = 0.05$; Figure A8c). We also detected a significant effect of time ($\chi^2 = 35.87$, df = 1, $p < 0.001$) where fish were closer to their neighbors in the morning compared to the afternoon. Our primary analysis of social cohesion (PC 2) revealed similar results, with fish showing tighter shoals in the stable and flux treatments compared to the clear condition. We further detected a significant effect of experiment ($\chi^2 = 13.40$, df = 1, $p < 0.001$) where fish were closer to their neighbors in the clay experiment compared to the dye experiment, which is also reflective of our PC 2 results.

For aggressive contacts, we detected a significant effect of treatment ($\chi^2 = 13.35$, df = 2, $p = 0.001$) where fish performed more aggressive contacts in the clear control and flux treatment compared to the stable treatment (*post hoc* pairwise comparisons: clear x stable, z-ratio = 2.45, df = ~Inf, $p = 0.04$; flux x stable, z-ratio = 3.71, $p = 0.001$; Figure A8d). These results are similar to our PC 3 results, where we observed that fish were more aggressive in the clear and flux treatments compared to the stable treatment. We also detected a significant effect of experiment ($\chi^2 = 5.59$, df = 1, $p = 0.02$) where fish performed more aggressive contacts in the dye experiment compared to the clay experiment.

For proximity to the nearest wall, we detected a significant interaction between treatment and experiment ($\chi^2 = 12.60$, df = 2, $p = 0.002$). In the clay experiment, fish were closer to the nearest wall in the stable treatment compared to the clear control and flux treatment (*post hoc* pairwise comparisons: clear x stable, t-ratio = 2.54, df = 213, $p = 0.03$; flux x stable, t-ratio = 4.15, df = 213, $p < 0.001$; Figure A8b). In our primary analysis of anxiety (PC 4), we found a similar significant interaction between treatment and experiment, though significance was lost during our *post hoc* analyses. We also detected a significant interaction between treatment and time ($\chi^2 = 10.50$, df = 2, $p = 0.01$). In the morning, fish

were also closer to the nearest wall in the clear control and stable treatment compared to the flux treatment (*post hoc* pairwise comparisons: clear x flux, t-ratio = -2.59, df = 213, p = 0.03; flux x stable, t-ratio = 3.26, df = 213, p = 0.004). This was again similar to our PC 4 results where fish showed more anxious behavior in the afternoon than in the morning.

Figure A8. Notable significant effects of (a) mean speed, (b) proximity to the nearest wall, (c) mean neighbor distance, and (d) aggressive contacts.

Box plot formatting is the same as in Figure A1. In Figure A8a and A8b the suspended sediment experiment (Experiment 1) is represented by shades of tan and the dye experiment (Experiment 2) is represented by shades of grey. Across all subfigures the treatment conditions are represented by the shade of the boxplot color, with the palest shade representing the clear control, the middle shade representing the flux treatment, and the darkest shade representing the stable treatment. Figure A8a shows the mean (cm/s) of fish during the morning recordings across both treatments and experiments. Figure A8b shows the proximity of the fish to the nearest wall (cm) across both treatments and experiments. Figures A8c and A8d respectively show the mean neighbor distance (cm) and the number of aggressive contacts performed by the fish across each treatment.

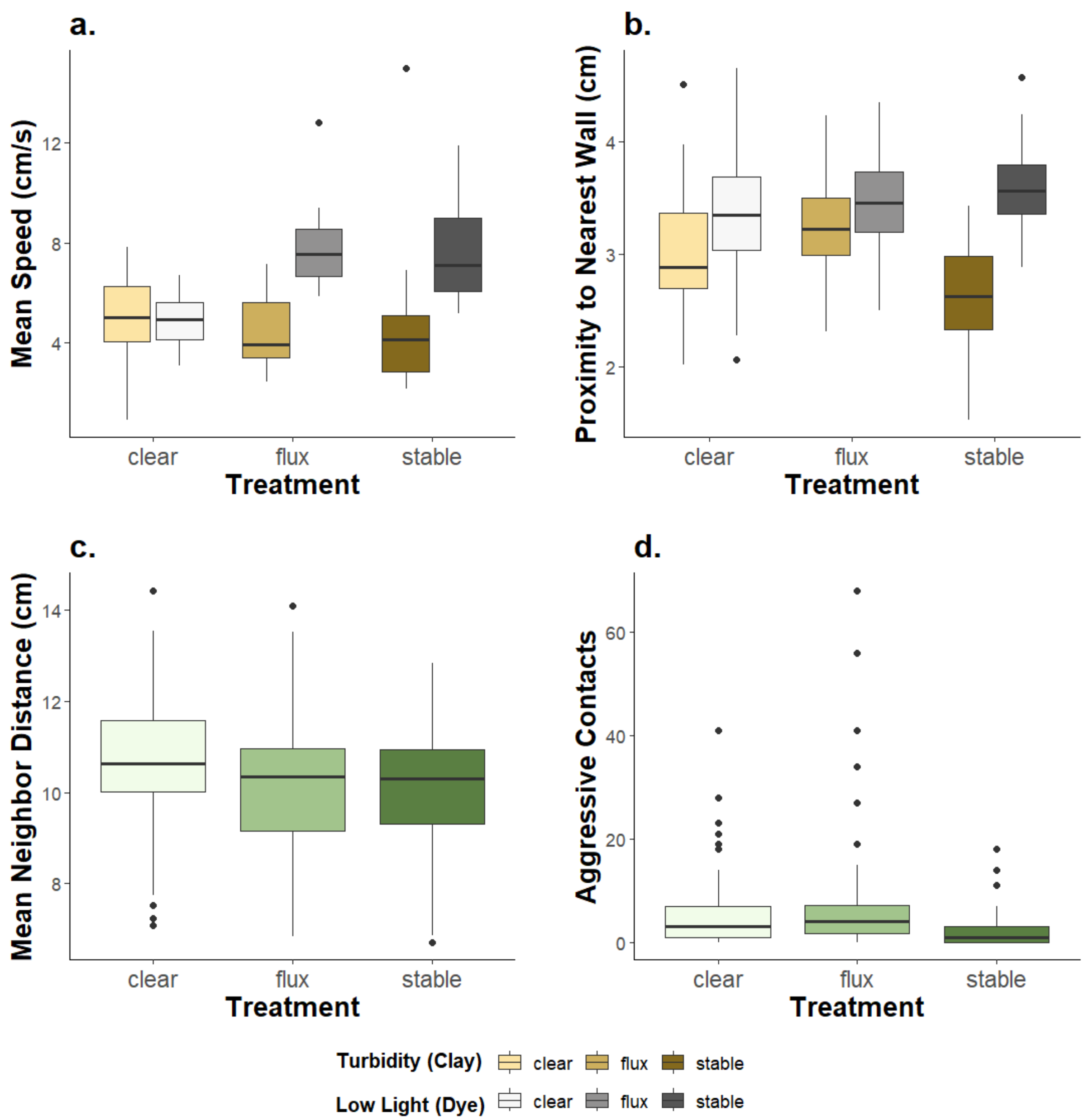

Supplement: Supplementary file 1 [file ECE3-16-e74157-s001.pdf]
